# History dependence in insect flight decisions during odor tracking

**DOI:** 10.1371/journal.pcbi.1005969

**Published:** 2018-02-12

**Authors:** Rich Pang, Floris van Breugel, Michael Dickinson, Jeffrey A. Riffell, Adrienne Fairhall

**Affiliations:** 1 Neuroscience Graduate Program, University of Washington, Seattle, Washington, United States of America; 2 Department of Physiology and Biophysics, University of Washington, Seattle, Washington, United States of America; 3 Division of Biology and Bioengineering, California Institute of Technology, Pasadena, California, United States of America; 4 Department of Biology, University of Washington, Seattle, Washington, United States of America; UNITED STATES

## Abstract

Natural decision-making often involves extended decision sequences in response to variable stimuli with complex structure. As an example, many animals follow odor plumes to locate food sources or mates, but turbulence breaks up the advected odor signal into intermittent filaments and puffs. This scenario provides an opportunity to ask how animals use sparse, instantaneous, and stochastic signal encounters to generate goal-oriented behavioral sequences. Here we examined the trajectories of flying fruit flies (*Drosophila melanogaster*) and mosquitoes (*Aedes aegypti*) navigating in controlled plumes of attractive odorants. While it is known that mean odor-triggered flight responses are dominated by upwind turns, individual responses are highly variable. We asked whether deviations from mean responses depended on specific features of odor encounters, and found that odor-triggered turns were slightly but significantly modulated by two features of odor encounters. First, encounters with higher concentrations triggered stronger upwind turns. Second, encounters occurring later in a sequence triggered weaker upwind turns. To contextualize the latter history dependence theoretically, we examined trajectories simulated from three normative tracking strategies. We found that neither a purely reactive strategy nor a strategy in which the tracker learned the plume centerline over time captured the observed history dependence. In contrast, “infotaxis”, in which flight decisions maximized expected information gain about source location, exhibited a history dependence aligned in sign with the data, though much larger in magnitude. These findings suggest that while true plume tracking is dominated by a reactive odor response it might also involve a history-dependent modulation of responses consistent with the accumulation of information about a source over multi-encounter timescales. This suggests that short-term memory processes modulating decision sequences may play a role in natural plume tracking.

## Author summary

Many important behaviors require animals to make extended decision sequences in response to complex stimuli. In this study we investigated the sequences of navigational decisions made by fruit flies and mosquitoes while tracking odor plumes, which are generally subject to turbulence. By examining video-taped 3D trajectories of these insects flying in a wind tunnel containing an attractive odor plume, we asked what features of encounters with the plume influenced odor-triggered flight decisions. Most notably, we found that although the average response was dominated by a reflexive upwind turn, its strength was modulated by the history of prior plume encounters throughout the trajectory. While no theoretical strategy we simulated captured all aspects of the data, the algebraic sign of the history dependence was only recapitulated in a model where a simulated tracking agent maximizes information about the position of the plume source. This suggests that real odor tracking may involve short-term memory processes over multi-encounter timescales that are consistent with the accumulation of information about source location.

## Introduction

In contrast to two-alternative laboratory decision-making paradigms, an animal making decisions in the natural world typically executes extended sequences of choices in response to stimuli with complex spatiotemporal statistics. One such natural decision process is the tracking of odor plumes to locate food sources and mates [1]. Natural odor plumes have complex distributions due to turbulence, which breaks plumes into intermittent filaments of odor interrupted by large areas of clean air [2, 3]. Thus, an insect’s navigational decisions during plume tracking must be made in response to sparsely distributed odor encounters that provide incomplete information about the source location [4]. At present, it is not well understood which features of in-flight encounters with a turbulent plume contain the most useful information about source position, nor how insects integrate these features for successful localization. Investigating this problem in flying insects will help illuminate this decision process and identify biologically relevant statistics of natural windborne signals. Ultimately, this may help in the development of strategies to combat insect-borne illness as well as inspire the design of robots used to track signals advected by fluids [5]. More generally, we hope our work will inform theories of the neuroscience underlying the accumulation of sensory evidence and how this evidence is used in active sensing and goal-directed decision making.

Because turbulent stimuli are difficult to both measure and control, experiments investigating search strategies during flight can only approximate naturalistic environments. This is frequently done using small plume-containing wind tunnels, in which the trajectories of flying insects are recorded with video cameras. Such studies showed, for example, that plume-tracking behaviors are highly influenced by the large-scale statistics of plume structure ([1]). When tracking CO_2_, for instance, mosquitoes exhibited increased upwind flight in a fluctuating, as compared to a homogeneous, plume [6]; further, continuous ribbon plumes elicited less upwind flight in moths than rapidly fluctuating plumes [7]. These both suggest that at least the coarse spatiotemporal structure of a plume affects tracking flight, although the mechanisms underlying this behavior remain unknown. It has also been suggested that the duration, concentration, and concentration gradient of odor packets may be informative plume features [8–13], but it is not known how these properties influence fine-scale navigational decisions or how these fine-scale decisions combine to form a global search strategy.

Fine-scale aspects of plume-tracking strategies have been primarily investigated in laminar flow (i.e., non-turbulent) wind tunnels with stationary plumes. Here, knowledge of the plume’s stationary profile allows one to infer the approximate plume concentration experienced by an insect based on its position at every moment in its flight trajectory. Subsequently, one can ask what behavioral responses are triggered by encounters with the plume. The current model for how flying insects track a source is that they follow a sequence of reflexes, in which they “surge” upwind upon entering the plume and “cast” across the wind upon leaving it. Some evidence of the surge-cast reflex strategy has been found in moths [14], in fruit flies tracking an ethanol plume [15], and in mosquitoes tracking CO_2_ [16]. A related but distinct “counterturning” (zigzagging) behavior, triggered by plume-crossing, has been observed in moths tracking pheromone plumes [7, 14, 17, 18].

**Fig 1:**
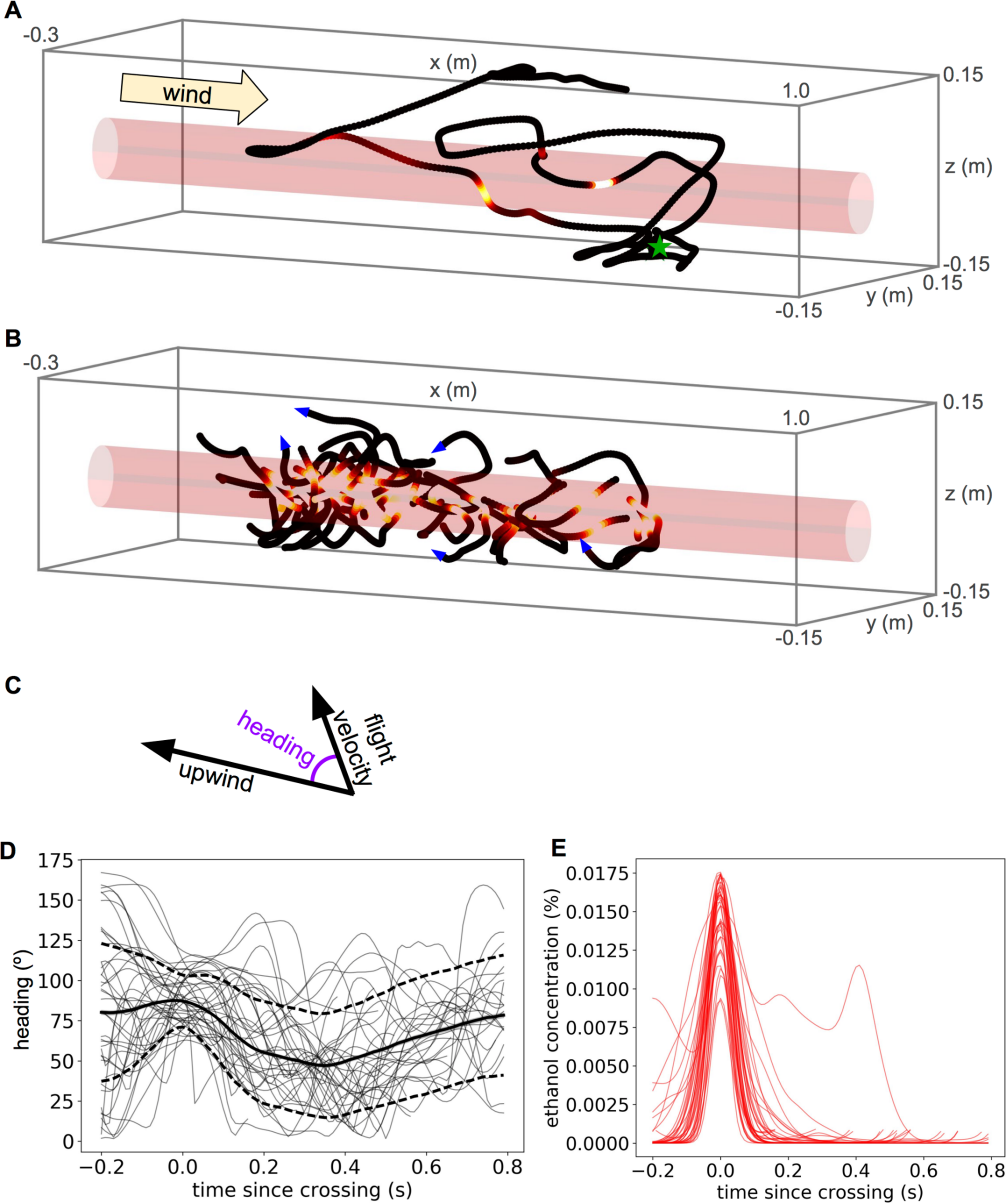
Example flight patterns and illustration of variability. A. Example fruit fly trajectory in a wind tunnel containing a stationary ethanol plume. The plume outline is shown in red, with the shell indicating 2 standard deviations from the plume’s centerline. The trajectory color shows the instantaneous odor concentration experienced by the fly, and the green star marks where the fly took off. B. Example plume-crossings from several trajectories. Blue arrows on a subset of crossings indicate the direction of flight. C. Heading is defined as the angle between the upwind direction and the fly’s velocity vector. D. Heading time courses surrounding example crossings (gray), as well as mean (black solid) and standard deviation (black dashed), relative to the crossing time. E. Odor concentration time courses surrounding example crossings.

While the surge-cast strategy is consistent with some aspects of plume tracking and provides an intuitive mechanism that can lead a simulated tracking agent to the source of a laminar plume [15], its most important shortcoming is that while it explains average behavioral responses to plume encounters, it does not explain the diversity of individual navigational decisions. Indeed, though the mean behavioral response of a fruit fly crossing an ethanol plume is to turn briefly upwind, individual responses vary over a range of nearly 100 degrees of turning angle (**Fig 1D**; the corresponding odor concentration timecourses for example crossings are shown in **Fig 1E**). In a similar analysis of moth flight trajectories, for example, investigating variability around the mean led to novel observations about moment-to-moment behaviors, such as ground speed variations in turning vs straight flight and decreases in ground speed upon approach to the pheromone source, which were not apparent from the mean responses alone [19]. What is not known is whether the diversity in fruit fly odor-triggered responses arises purely from unobservable variations in small air currents or the insect’s internal state, or whether some of it can be predicted from measurable stimulus features. In the context of courtship, for instance, an analysis of behavioral diversity indeed showed that much variability in fruit fly singing previously assumed to be random could in fact be explained by variations in the fly’s recent sensory experience [20], which allowed the authors to better hypothesize about the underlying neural mechanisms.

Here, we explore the sources of diversity in plume-tracking-related flight maneuvers by analyzing a dataset of fruit fly (*Drosophila melanogaster*) and mosquito (*Aedes aegypti*) flight trajectories through a wind tunnel containing a stationary odor plume, and by asking which detectable features of odor encounters systematically modulate flight maneuvers. We find that fruit flies, and possibly mosquitoes as well, turn more strongly upwind in response to higher experienced concentrations, and that later encounters in a sequence of encounters along a trajectory trigger weaker upwind turns. To our knowledge, the latter result provides the first evidence for information accumulation over multiple plume encounters in in-flight plume-tracking in fruit flies and mosquitoes. This suggests that purely reactive models of insect plume-tracking might be significantly augmented by a history dependence term in which upwind flight slows as more encounters occur.

## Results

We analyzed datasets of fruit fly and mosquito flight trajectories recorded with a camera-based tracking system as they interacted with either an ethanol (fruit flies) or carbon dioxide (mosquitoes) plume. The data were originally described in van Breugel and Dickinson [15] (fruit flies) and van Breugel et al. [21] (mosquitoes). Briefly, a low velocity non-turbulent wind flowed along the long axis of a wind tunnel in the center of which was a stationary laminar odor plume (**Fig 1A and 1B**). The plume had an approximately Gaussian cross-sectional profile, with maximum concentration along the centerline, and the floor of the wind tunnel contained a checkerboard pattern to provide the insects with a visual cue by which to orient. Insects were allowed to explore the wind tunnel for several hours, and whenever they took off, their trajectories were captured by the tracking system until they landed. The 3D position sequence of the trajectory was reconstructed in real time from the videos. Because the plume was stationary with a previously measured concentration profile, the dynamically varying odor concentration experienced by the insect at each time point could be estimated from its position. To understand how specific plume crossing features influenced decisions, we investigated the time-varying heading (where heading is defined as the angle between the fly’s velocity vector and upwind [**Fig 1C**]), time-locked to the time of the peak concentration experienced during the plume crossing (**Fig 1B, 1D and 1E**). We defined a plume crossing as the portion of a trajectory when the odor experienced by the fly exceeded a minimum concentration threshold. Our criteria for choosing this threshold are discussed in *Methods*, but the conclusions that follow are not strongly affected by its precise value.

**Fig 2:**
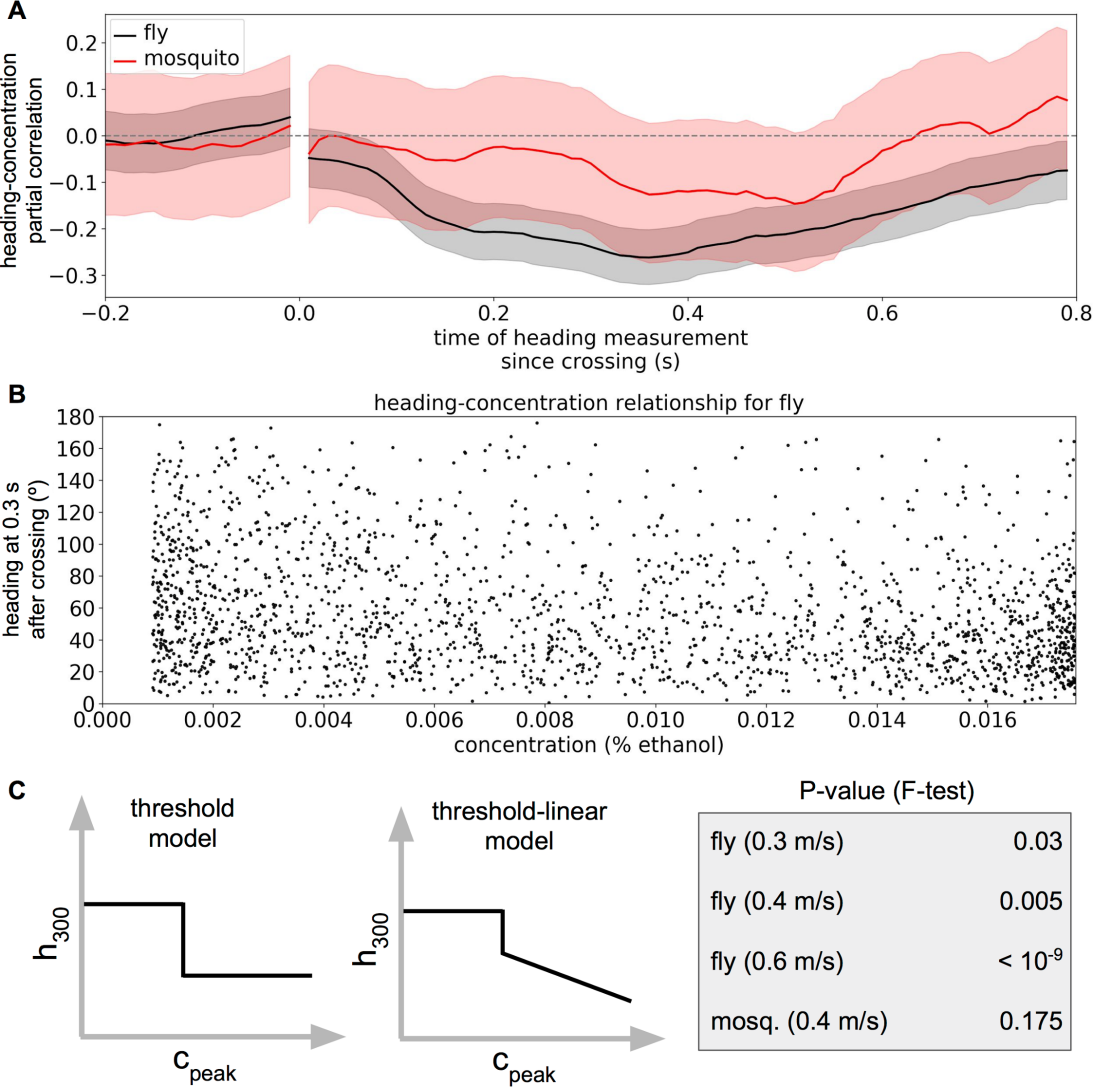
Dependence of heading on peak concentration. A. Partial correlation of peak odor concentration experienced by the animal and subsequent heading at various times past the odor peak for fruit flies and mosquitoes. Shading indicates 2.5–97.5% confidence interval. B. Heading at 300 ms since odor concentration peak as a function of peak odor concentration during crossing. Each point represents a single plume-crossing. Though a significant correlation exists, the joint distribution is dominated by variability. C. Threshold and threshold-linear models for identifying non-binary dependence of h_300_ (the heading 300 ms post-crossing) on concentration. For mosquitoes, h_500_ (the heading 500 ms post-crossing) was used. P-values indicating the probability that the threshold-linear model would have fit better by chance (F-test) are shown in the gray box for four different experiments (fruit flies following an ethanol plume in 0.3, 0.4, or 0.6 m/s winds, and mosquitoes following a CO_2_ plume in 0.4 m/s wind).

Because natural flight trajectories typically cross the odor plume repeatedly, each time yielding a potentially unique sensory experience, we first asked whether the crossing-triggered flight maneuvers depended on the peak concentration experienced during the crossing. We need, however, to control for effects of the animal’s initial heading and location in the tunnel. To do this, we calculated the partial correlation between peak experienced odor concentration and the insect’s heading at different points in the near future, conditional on *x*_0_, the insect’s position along the long axis of the wind tunnel, and *h*_0_, its initial heading at the time of the crossing. This conditioning allowed us to subtract out any linear dependence on these purely geometric factors. To further isolate the concentration-dependent effects from those deriving from initial location or heading angle, in all subsequent analysis we include only crossings occurring in the middle portion of the wind tunnel (i.e., not in the upwind- or downwind-most 30 cm) and only crossings for which the insect crossed the plume such that *h*_0_ was between 60° and 120°. This selection of the crosswind heading range was motivated by the canonical view that plume crossings on average trigger upwind turns [15, 16], which would be more apparent if the insect were not headed upwind already. As shown in **Fig 2A** and **2B** for fruit flies we found a significant negative partial correlation between the peak concentration experienced in the crossing and their flight heading 300 ms later, suggesting that higher concentrations elicit stronger upwind turns.

A correlation between peak concentration and subsequent heading could also arise, however, if the response were purely binary but noisy—as has been implicitly assumed in previous odor-tracking investigations [15] as well as in theoretical models for tracking turbulent signals [22]. To test whether, in contrast, heading may depend continuously on odor-encounter concentration, we compared two simple models predicting *h*_300_, (the heading 300 ms post-crossing, or 500 ms [*h*_*500*_] for mosquitoes), from *c*_*peak*_, the peak concentration experienced in the crossing preceding the heading measurement (**Fig 2C**). In the *binary* model, *h*_300_ was assumed to take one mean value when *c*_*peak*_ was below a threshold *c*^*th*^ and another when *c*_*peak*_ was above *c*^*th*^, with the linear effects of *h*_0_ and *x*_0_ again subtracted out to minimize geometric confounds. We compared this with a *threshold-linear* model, in which *h*_300_ was assumed to be a linear function of *c*_*peak*_ when *c*_*peak*_ was greater than *c*^*th*^. The threshold-linear model allowed us to retain the possibility that sufficiently weak concentrations are not detectable, without having to assume that this would be accounted for by a linear prediction. We fit both models by minimizing the squared prediction error of *h*_300_. We found that for fruit flies the threshold-linear model fit significantly better than the binary model (F-test, with p-values shown in **Fig 2C**), corroborating our hypothesis that behaviorally relevant concentrations are not processed in a simple binary way during in-flight plume tracking. For mosquitoes, there was not enough data to see a significant difference between the fits (p = 0.175) (**Fig 2C**).

Beyond purely reflexive flight maneuvers, recent theoretical results suggest that more efficient source localization can result from integrating information from multiple plume encounters over an extended period of time [22, 23]. Such information integration should lead to a dependence of crossing-triggered flight maneuvers on trajectory history. To explore this, we asked whether it is possible to distinguish flight maneuvers that are triggered by later compared with earlier plume crossings in an extended sequence of crossings.

**Fig 3:**
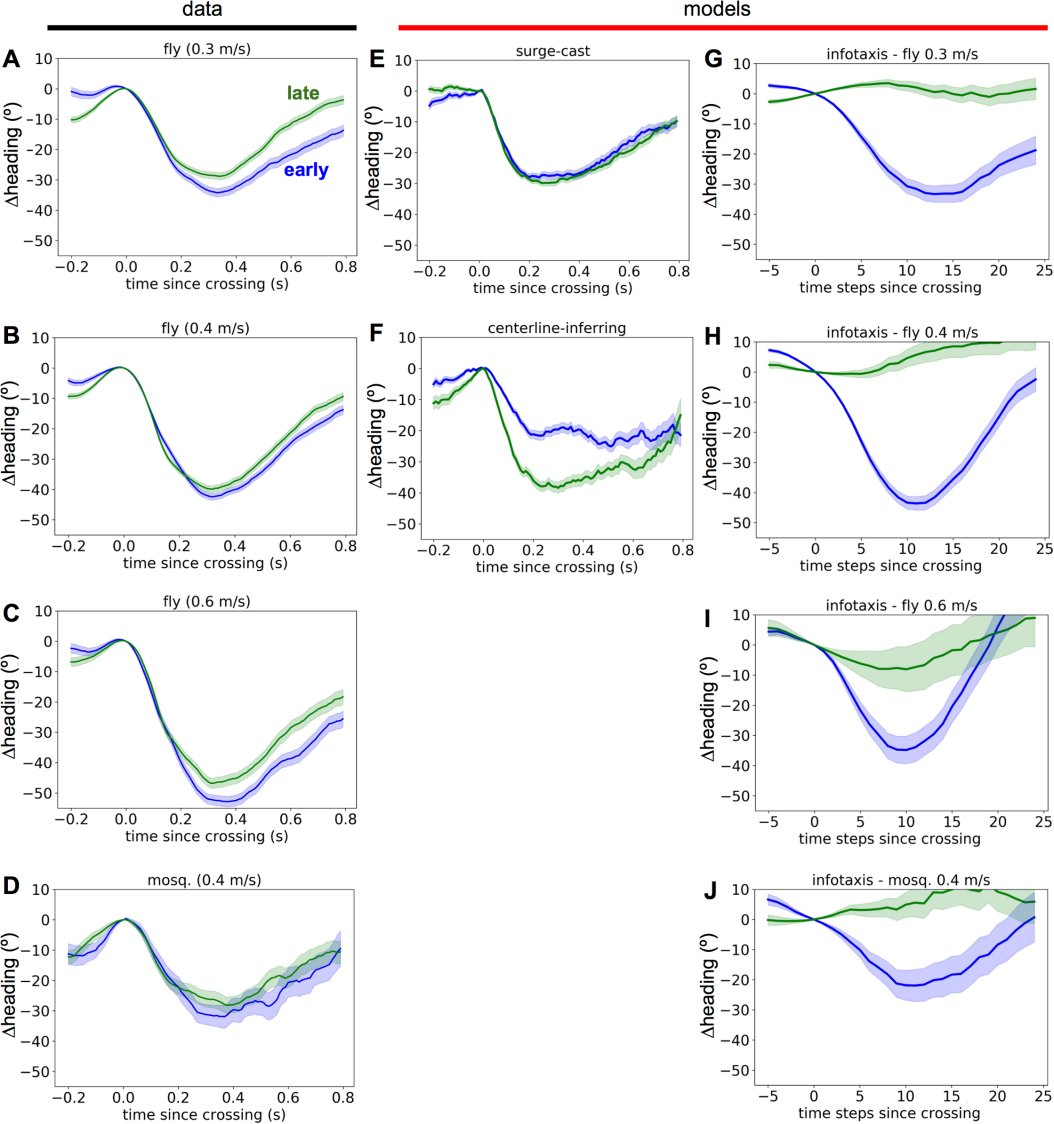
History dependence of crossing-triggered turns in data and models. A-D. Crossing-triggered heading time courses for early (blue) or late (green) crossings for fruit flies tracking an ethanol plume in three different wind speeds, or mosquitoes flying in a wind tunnel with a 0.4 m/s wind. Thick lines indicate means, with shading indicating standard error of the mean. E. Same as A but for trajectories generated using the surge-cast model. F. Same as A but for trajectories generated using the centerline-inferring model. G-J. Same as A-D but for trajectories generated using the infotaxis algorithm, with wind speeds and plume profiles matched to each of the four experiments.

When we separated plume crossings into *early* (the first or second in a trajectory) vs. *late* (the third or later in a trajectory) crossings, we found that while in both cases, the average flight is strongly dominated by a brief upwind turn, late crossings tended to trigger weaker upwind turns than early crossings (**Fig 3A–3D**). **Fig 3E–3J** show the equivalent crossing distributions for three different models, one reactive (surge-cast [**Fig 3E**]) and two whose behavior depends on information about the plume accumulated over time (centerline-inferring [**Fig 3F**] and infotaxis [**Fig 3G–3J**]), which are discussed in more detail later in the text. There may be geometric confounds to identifying history dependence in the data: later crossings may generally occur in the more upwind section of the wind tunnel since the insect has made more upwind progress, which might result in more crosswind turns simply because the insect has a smaller area to turn into. However, we found that the position along the long axis of the wind tunnel at the time of crossing, *x*_0_, differed by less than 3 centimeters between early and late crossings (about 2% of the total wind tunnel length) (**S2 Fig**).

**Fig 4:**
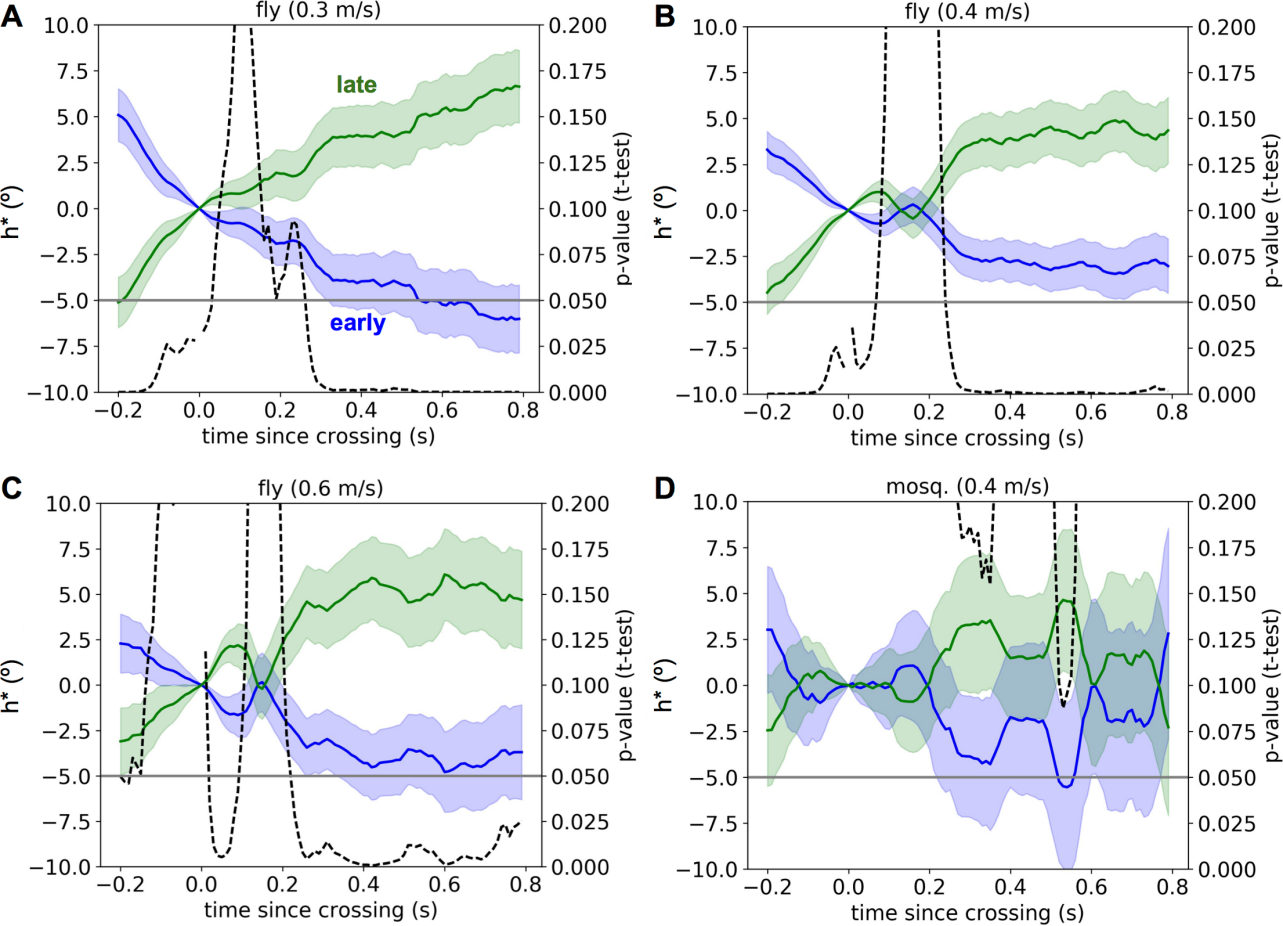
History dependence of post-crossing turns after accounting for position and flight time. Each panel shows the mean and standard error of the mean (shading) of h*(t) across different time points post-crossing for early (blue) and late (green) crossings. The dashed line indicates the probability that such a difference in means would have arisen from chance, with the gray line marking 0.05. A. Fruit fly experiment in 0.3 m/s wind (783 early crossings, 766 late crossings). B. Fruit fly experiment in 0.4 m/s wind (1158 early, 828 late). C. Fruit fly experiment in 0.6 m/s wind (394 early, 294 late). D. Mosquito experiment in 0.4 m/s wind (125 early, 143 late).

To treat potential confounds, not only geometric but also those possibly due to fatigue from extended flying (e.g. flies that have been flying longer may choose more crosswind paths to save energy), we defined a new heading change variable h*(*t*) at each time point post-crossing, computed as the original Δ*h*(*t*) with the best-fit linear prediction from *x*_*0*_ and *T*, the total flight time since take-off, subtracted out, thus removing any linear dependence on these variables of whether the crossing was early vs. late. As shown in **Fig 4**, the early vs. late time courses of h*(t) were significantly different from about 300 ms post-crossing onwards for all of the fruit fly experiments, with late heading changes being more crosswind than early heading changes. We also calculated the partial correlation between the heading change, time-averaged from 350 ms to 450 ms post-crossing, and the crossing number, conditioned on *x*_0_ and *T*. Note: these plots appear symmetrical about h*(*t*) = 0° because the mean Δ*h*(*t*) across all crossings is recomputed and subtracted out during the linear fitting at each timepoint. Thus, h*(*t*) averages to 0° by construction. Since there are similar numbers of crossings in each group, and since the early crossings have stronger upwind components (h*(*t*) < 0), whereas the late crossings have stronger crosswind components (h*(*t*) > 0), the average within-group h*(*t*)’s end up about equidistant from 0°. The heading change and crossing number were significantly correlated even after removing all correlations through *x*_0_ and *T* (**S4 Fig**). These two analyses provide further evidence that the small history dependence in the fly’s trajectories was due neither to the geometry of the wind tunnel nor to fatigue of the insects, but was instead related to their extended interactions with the plume. Additionally, while increased wind speed across the fruit fly experiment tended to yield stronger upwind turns overall, as shown in **Fig 3A–3C** and **Fig 4A–4C** it did not appear to have any consistent effect on the history dependence.

**Fig 5:**
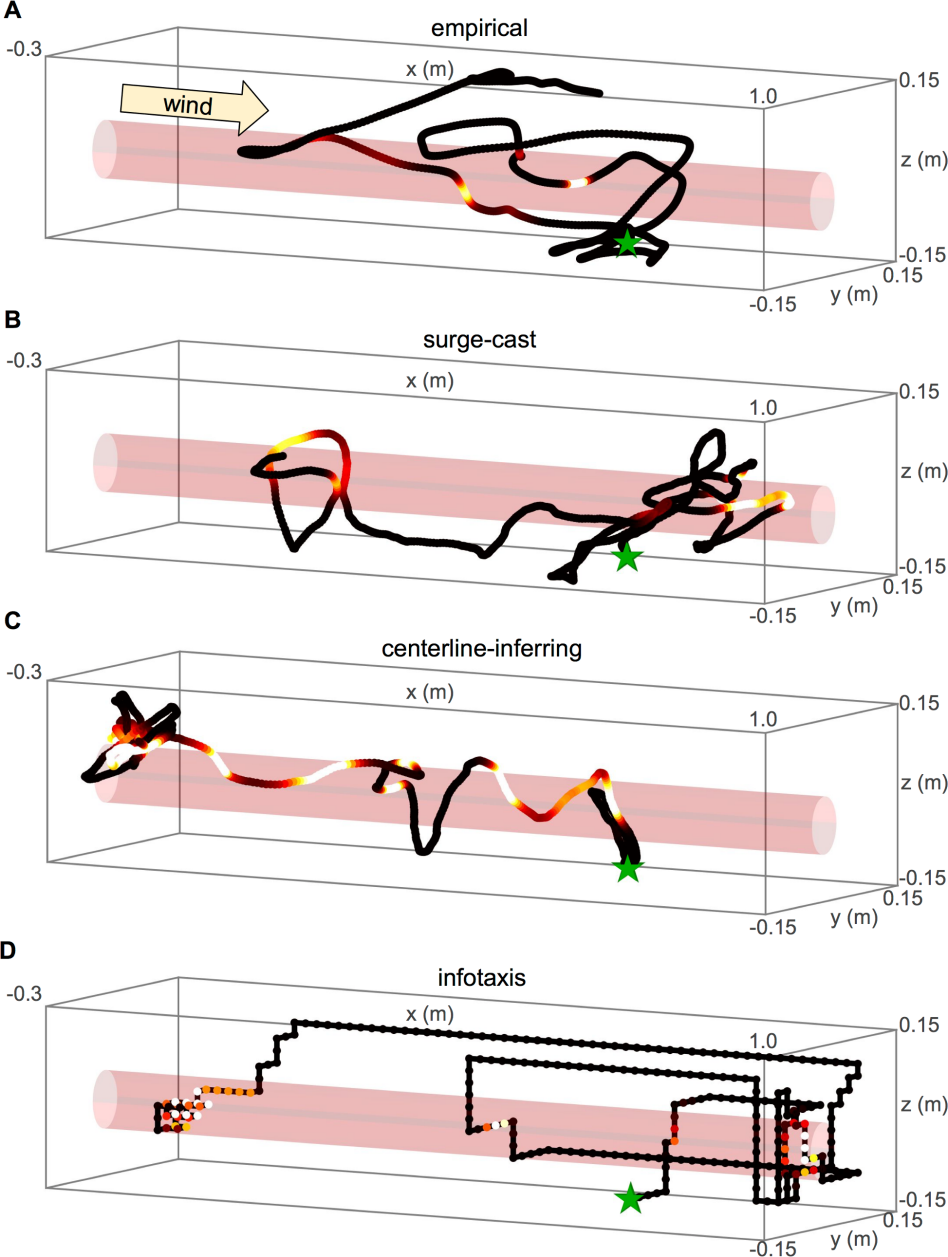
Example empirical and simulated trajectories. Each of the simulated trajectories (B-D) began at the same location in the wind tunnel (green star) as the empirical trajectory (A) and was run for the same length of time as the empirical trajectory.

How does the history dependence in plume-crossing-triggered flight responses compare with that predicted by theoretical algorithms? To address this we considered three plume-tracking strategies capable of generating complete flight trajectories, in a simulated wind tunnel containing a plume with the same geometry as that in the experiments. We first considered a purely reflexive “surge-cast” algorithm, based on the model proposed by van Breugel and Dickinson [15]. Here, however, we initially modeled the insect to have baseline flight dynamics given by a correlated random 3D walk with a moderate bias towards casting (crosswind flight), with parameters fit to match the insects’ empirical flight speed, angular velocity, and crosswind position distribution as best as possible (**S6 Fig**). In addition to the random walk dynamics, whenever the insect crossed the plume, it was subjected to a brief upwind “surge” force, modeled as an alpha function with a time constant of 0.07 s. An example empirically observed trajectory is shown in **Fig 5A**, with a trajectory generated by the surge-cast model shown in **Fig 5B**. **Fig 3E** shows that the surge-cast model recapitulates the dominant upwind component of crossing-triggered turns but does not yield an observable distinction between early and late crossings. This shows that the reflexive maneuvers generated by this model are not sufficient to yield the history dependence we observed in the data.

An alternative model suggests that as an insect should bias its flight more strongly upwind as it gains information about the location of the plume centerline, that is, it should slowly switch from an explorative crosswind to an exploitative upwind strategy as it learns where the plume is located [23]. This model allows for the probability distribution over the source’s centerline to be updated in a Bayesian way with only simple arithmetic computations. Instead of using Grunbaum’s model directly, in order to study it we applied its general “centerline-inferring” principle to our baseline correlated random 3D walking model: instead of reflexively surging upon crossing the plume (as in the surge-cast model), whenever this modeled insect crossed the plume at a certain location, it updated the mean and uncertainty of its estimate of the plume’s centerline position according to Bayes’ rule. An additional biasing term then drove the insect upwind, with a magnitude that increased as the uncertainty of the centerline estimate decreased. We refer to this as the “centerline-inferring model”, an example trajectory of which is shown in **Fig 5C**. We additionally included a finite memory timescale, allowing the estimate over the plume centerline to decay back to its baseline over the course of about 10 seconds.

The history dependence of trajectories generated by the centerline-inferring model was reversed compared to the empirical data (**Fig 3F**). Specifically, late plume crossings yielded stronger upwind turns than early plume crossings, suggesting that this model likely cannot account for the sign of the history dependence observed in empirical trajectories. Note that neither the surge-cast nor centerline-inferring models used wind speed as a parameter, since flight velocities were calculated relative to the environment. While it would have been possible to include such a parameter in the models so that we could reproduce, say, the stronger upwind turns in higher winds (**Fig 3A–3C**), our goal here was to study history dependence, which was not systematically affected by wind speed, and so we excluded it to focus and simplify our model.

The final plume-tracking strategy we investigated, which is more theoretically principled but also more cognitively demanding, was “infotaxis”, an algorithm proposing that a tracking agent should move to maximally decrease the uncertainty over possible source locations in 3D space, with the specific form and parameters of the distribution derived from the statistics of turbulence [22]. An example trajectory is shown in **Fig 5D**. Though the “true” plume used in our infotaxis simulations was cylindrical, as in the experiments, it is important to note that the tracking agent’s internal model is of a turbulent one. That is, we simulated how an agent used to tracking natural, turbulent plumes, would respond when introduced to a laminar one, a paradigm we thought best reflected the situation in the experiments. As with the centerline-inferring model, we compared infotaxis to our data by examining the history dependence in early vs. late crossing-triggered heading time-series in a set of infotaxis trajectories generated within a simulated plume-containing wind tunnel. The wind speed parameter, which influences the agent’s internal plume model, we set equal to that from each experiment, assuming that an ideal agent would be able to measure the wind speed. We chose the coefficient of turbulent diffusivity, the principal parameter in infotaxis governing how far a tracking agent thinks the plume would naturally spread from a point source, so as to roughly reflect the length scale of the wind tunnel and the assumption that fluctuations in a non-laminar flow would be at about the same scale as the mean wind speed itself.

The infotaxis trajectories resembled the empirical data in the sign of the history dependence observed: late plume crossings in infotaxis trajectories tended to yield turns with a weaker or completely absent upwind component than early plume crossings (**Fig 3G–3J**), as in the real data (**Fig 3A–3D**). The magnitude of the odor-triggered turns, however, was quite different, suggesting that infotaxis does not fully capture all aspects of the plume crossing distribution. Nevertheless, this suggests that the true plume-tracking algorithm used by fruit flies shares certain features with infotaxis that cannot be captured by other normative tracking models.

**Fig 6:**
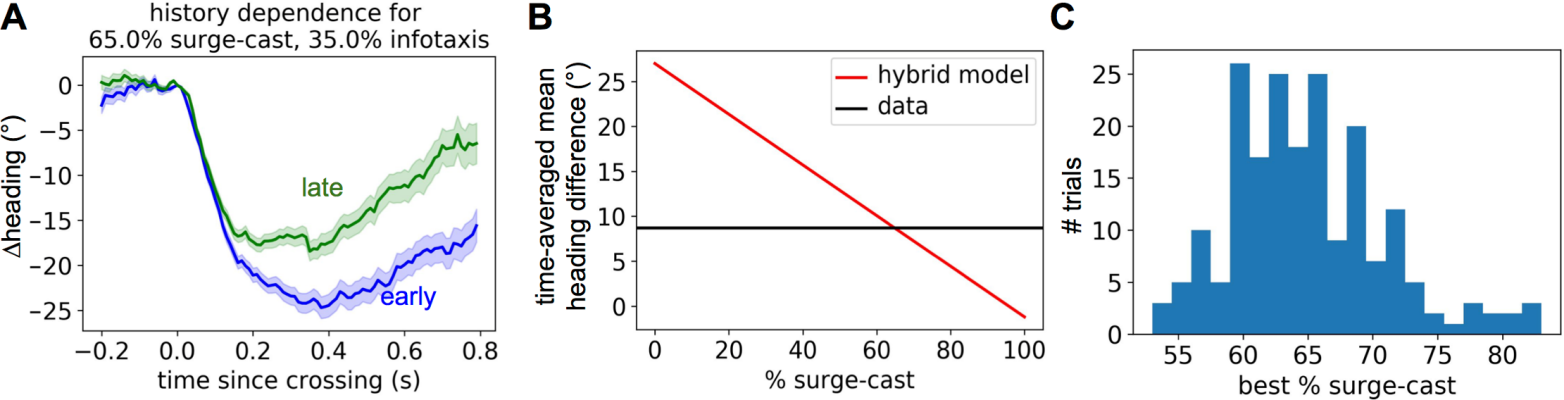
Analysis of hybrid surge-cast-infotaxis crossing model. A. Hybrid surge-cast-infotaxis crossings, as described in text, separated into early vs. late crossings with a crossing model mixing 65% surge-cast with 35% infotaxis. B. The late vs. early difference in mean headings of the hybrid crossings (time-averaged from 0.3 to 0.8 s post-crossing) as a function of percentage of surge-cast making up the model (red), overlaid with that calculated from the 0.3 m/s wind speed fruit fly experiment (black). C. The distribution of data-matched surge-cast % over multiple random pairings of surge-cast and infotaxis crossings.

To quantify how much a simpler model, such as the surge-cast algorithm, would have to be modified by an infotaxis-like model in order to recapitulate the magnitude of the history dependence observed in the data, we also investigated a hybrid model of crossing-triggered heading responses. Here, a new set of crossings was built using weighted sums of the surge-cast and infotaxis crossings. Specifically, for each infotaxis crossing we selected a random surge-cast crossing with the same crossing number (i.e. we matched a third crossing from an infotaxis trajectory with a third crossing from a surge-cast trajectory), and then generated a new hybrid crossing composed of P% of the surge-cast crossing and (1-P)% of the infotaxis crossing, i.e., Δh^hybrid^(t) = P/100 x Δh^surge-cast^(t) + (1-P)/100 x Δh^infotaxis^(t). As with the other models, we then grouped the hybrid crossings into early vs. late categories and compared them quantitatively (**Fig 6A**). When we optimized the mixing percentage P to match the data, we found that the early vs. late crossing-triggered heading response could be best reconstructed with P equal to approximately 70% (**Fig 6B and 6C**), suggesting that an infotaxis-like history dependence gives about a 30% contribution to explaining the full crossing-triggered heading response.

**Fig 7:**
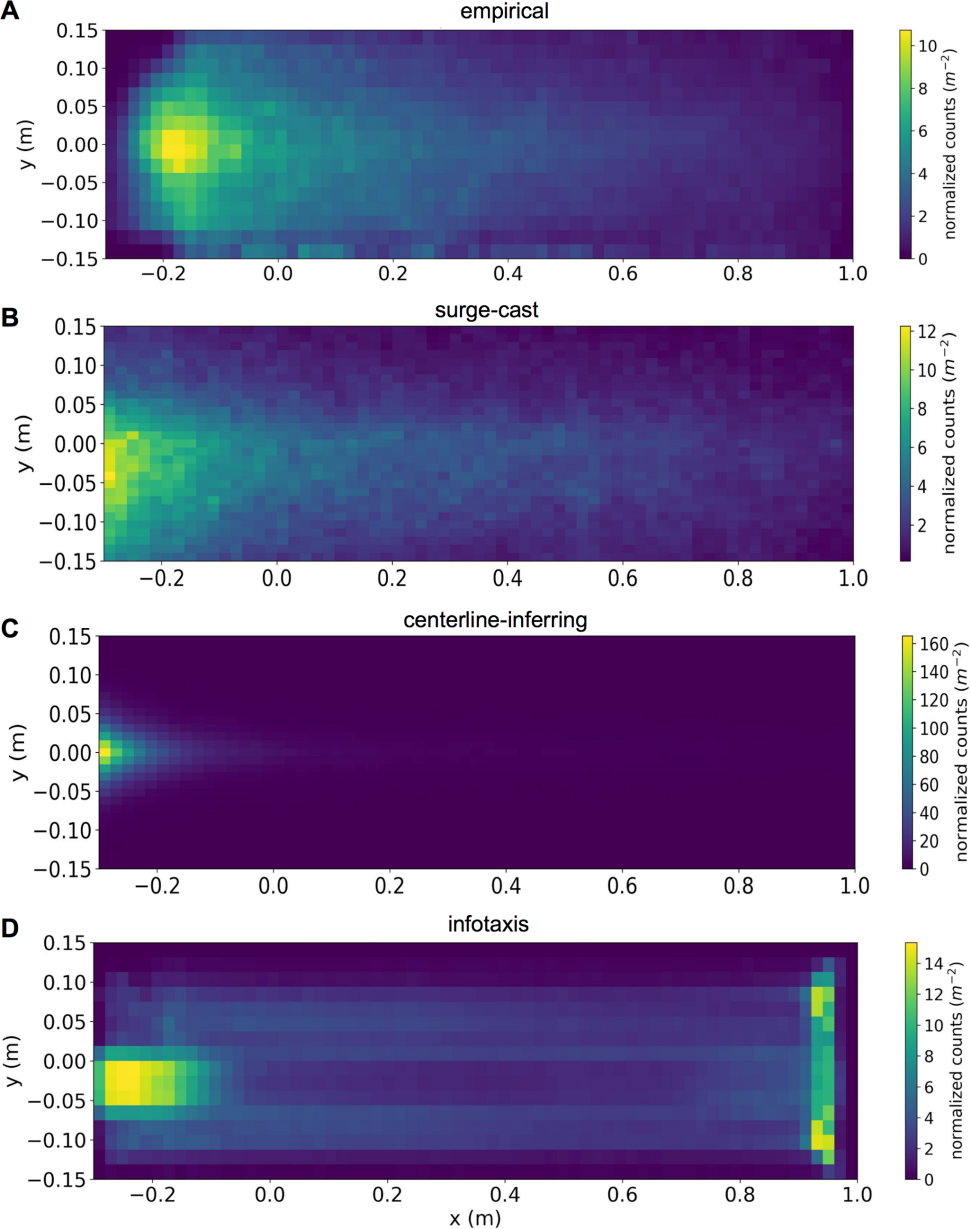
Trajectory heatmaps for data and models. Each distribution was calculated by binning all timepoints generated from all trajectories, empirical or modeled, used in our analyses. In this figure we have not excluded any timepoints based on position in the wind tunnel or heading. A. Empirical trajectories from fruit flies in a wind tunnel with an ethanol plume and 0.3 m/s wind. B-D. Equivalent trajectories generated by the surge-cast (B), centerline-inferring (C), or infotaxis (D) algorithms.

To show that infotaxis reproduced the sign of the empirically observed history dependence but not necessarily other qualities of the data, we compared the distribution of positions within the wind tunnel generated by either the real trajectories or those simulated by infotaxis, as well as by the two other models. Notably, the position distribution of the empirical trajectories exhibited a salient peak at the upwind end of the wind tunnel (**Fig 7A**, reproduced roughly from [15]). This was true for the surge-cast and centerline-inferring simulations as well (**Fig 7B and 7C)**. Notably, while the infotaxis simulation also yielded a salient peak in the upwind end of the wind tunnel, it exhibited an additional peak at the very downwind end of the wind tunnel (**Fig 7D**). This is likely due to the fact that in infotaxis it is usually preferable to initially travel to the downwind end of the wind tunnel in order to increase the probability of first encountering an odor hit. However, we note that in the infotaxis simulations corresponding to the 0.4 m/s and 0.6 m/s wind speeds, the strong peak in the upwind end of the wind tunnel was much less pronounced (**S8 Fig**). This, combined with the lack of a quantitative match between the crossing-triggered heading time courses in the data vs. in infotaxis, suggests that although infotaxis is able to capture certain features of the empirical data there are other key features that it fails to reproduce.

## Discussion

To explore how animals enact sequences of decisions in response to complex stimuli in a natural context, we examined flying insects tracking odor plumes and asked whether deviations from the mean odor-triggered responses could be predicted from specific features of the odor encounters. We found that higher concentrations of odor led to stronger upwind turns, and that encounters occurring later in a sequence triggered weaker upwind turns. While a reactive surge-cast strategy predicted the mean odor-triggered upwind turn well, only trajectories generated by the infotaxis strategy [22] showed consistency with sign of the observed history dependence, though the magnitude of the history dependence in infotaxis was greatly exaggerated relative to that of the data. This suggests that sequential decision processes in real plume tracking may be dominated by reactive responses but may also be modulated by information about a source location accumulated over the course of a flight trajectory.

### Measuring and controlling turbulent signals

Although it is relatively straightforward to reconstruct natural flight trajectories using videos, precise spatiotemporal patterns of a naturalistic odor signal are challenging to measure, since odor is usually advected by turbulent flow [24]. Instead one often measures the coarse-grained statistics of a turbulent signal, such as its time-averaged concentration, intermittency, or plume envelope, foregoing the ability to infer the odor concentration at precise timepoints, but allowing one to explore how trajectory features depend on these statistics [6, 7]. Alternatively one can present a stationary plume with a pre-measured profile, which allows one to infer the concentration experienced by a flying insect on a moment-by-moment basis, but in which case the true signal is less naturalistic. In this analysis we have examined flight trajectories generated in the latter context, since it allowed us to infer the moment-by-moment sensory experience of each insect and to look for correlations with subsequent behaviors. Further, from the perspective of the fly, which follows a highly variable path through the wind tunnel, its encounters with the plume may be a sufficient approximation to the encounters it would have with a turbulent plume [15]. Importantly, although the plume itself is stationary, the variation in each trajectory yields variation in the sensory experience of each fly. This was key in allowing us to explore how different features of plume encounters elicited different navigational decisions.

### Informative features of plume encounters

Our first result that flies respond to differences in the concentration experienced during an in-flight odor encounter suggests that this feature may contain usable information about the fly’s current position relative to the plume source. Indeed, it has been shown theoretically that the distribution of nonzero instantaneous concentrations depends on displacement from the plume source [13], and our results propose that flies detect and act on this information. This suggests that theoretical trajectory-generating plume-tracking strategies, such as infotaxis [22], should incorporate non-binary odor detections in order to better recapitulate observed animal trajectories; whether such information significantly improves tracking efficiency, however, remains an open question. For example, it may be the case that higher instantaneous concentrations indicate to the fly that it is closer to the plume centerline, such that it would behoove it to make a stronger upwind turn, as we observe in the data. Our result also corroborates the fact that flies have neural mechanisms capable of detecting graded levels of odor concentration, which have been observed at the level of ORNs [25, 26], but which may also be present in the antennal lobe, one synapse downstream from the detector neurons.

Our second result, that the history prior to an odor encounter influences a fly’s response to that encounter, suggests that the number of encounters throughout a naturalistic trajectory also contains information about the fly’s displacement from the source. For example, our observations are consistent with the possibility that increased encounters might indicate that the fly has moved closer to the source, such that it should diminish its upwind turns so as not to overshoot it. Indeed, such a strategy would be consistent with moth plume tracking behavior, in which it was seen that moths tended to slow their flight as they approached a pheromone source [19]. Alternatively, as the flies accumulate odor encounters they might be more inclined to investigate the visual features of the surrounding wind tunnel, such as the floor pattern [15], which might slow their upwind progress.

More generally, the dependence of the flight trajectories on odor history is consistent with the flies’ acting on information obtained from multiple, temporally separated samples of the plume, an idea fundamental to strategies based on extended accumulation of information [22, 23]. The neural mechanisms enabling history-dependent behaviors in plume tracking are unknown, but a simple possibility is that they result from adaptation of the peripheral olfactory neurons (ORNs) in the insects’ antenna and maxillary palp, which typically decrease their sensitivity after extended odor exposure [27, 28]. ORN adaptation is typically studied, however, with much longer pulses than the sparse, intermittent puffs expected in a turbulent plume, so it is unclear how strongly this mechanism would influence natural tracking. An alternative is that history dependence arises from state changes in the antennal lobe (AL), the insect olfactory network immediately downstream of the initial receptors, analogous to the mammalian olfactory bulb. Indeed, many local neurons within the *Drosophila* AL substantially change their odor-evoked responses over the course of an approximately 10 second train of short pulses, with some exhibiting decreased, and others increased responses to later pulses in the train [29]. Interestingly, these AL response dynamics occur on about the same timescale as the history dependence we have observed from in the behavioral analysis. Determining the precise neural mechanisms underlying our results will be an exciting future task, and this suggests that exploring antennal lobe mechanisms may be a promising avenue for investigation.

An important factor in experiments involving flight trajectories through wind tunnels is the visual experience of the insect. Since wind tunnels are typically small due to technical constraints, i.e., between 1–2 m long and between 0.3–1 m wide, such that the insects can likely see the boundaries of the environment, it is likely the case that flight decisions are driven by a combination of both visual and olfactory stimuli. Indeed, the optomotor response of flying insects requires visual input in order for the flies to determine the wind direction relative to the ground [1], so that experiments cannot be performed in the absence of visual input. In our analyses we have attempted to control for visual effects in several ways. Since the main effect we expect is for the fly’s distance to the upwind or downwind walls to influence their propensity to turn upwind (for example, they may be less inclined to turn upwind if there is little space for them to turn into), in our analyses of the empirical data we excluded plume crossings occurring in the upwind-most and downwind-most 30 cm of the wind tunnel. As an additional control, however, in each of our data analyses we subtracted out the best linear prediction of the quantity of interest given the position *x*_0_ of the fly along the upwind-downwind axis of the wind tunnel. Specifically, in quantifying the concentration dependence of plume-crossing-triggered heading, we first subtracted the best prediction of heading from *x*_0_ and then calculated the correlation of the residual heading variation with plume-crossing concentration, with a similar analysis applied to the dependence of plume-crossing-triggered heading on the number of prior crossings in the trajectory. In the future, it will be useful to conduct plume-tracking experiments in larger wind tunnels, such that the visual experience does not substantially vary across different locations in the wind tunnel, but doing so at present remains a significant technical challenge.

### Identifying evidence of theoretical search strategies in data

There has been much interest in elucidating theoretical principles of how insects track turbulent plumes [2]. However, due to the highly dynamical nature of flight trajectories, identifying evidence of specific search strategies in data is not straightforward. In particular, because the dynamical odor experience of each insect is controlled not by the experimenter but rather by the insect’s own movements, one cannot compare theoretical vs. empirically observed trajectories on a one-to-one basis. More precisely, one cannot force a simulated trajectory to have a data-matched odor experience without causing it to deviate potentially quite strongly from the algorithm that was supposed to have generated it.

Given these limitations, it is preferable to instead ask how the distributions over specific trajectory features vary between simulations and data. Since a key theoretical question asks about the extent to which insects accumulate and use information about the plume’s source location over extended time periods, we chose to ask how such information accumulation might affect the history dependence of navigational decisions observed in real trajectories vs. those simulated by theoretical algorithms. Consequently, we examined the joint distributions of plume-crossing-triggered heading responses and a variable indicating whether each plume crossing occurred early vs. late in the total sequence. Our findings were that although plume-crossing-triggered flight maneuvers were dominated by a brief upwind turn (**Fig 1D**), they indeed differed early or late in the trajectory (**Fig 3A–3D, Fig 4**). This suggests that although the real plume-tracking strategy used by the insects we explored likely involves a significant reactive component, it is also modulated by the insect’s history with the plume since it took to the air. This rules out plume-tracking strategies that are 100% reactive, such as the pure surge-cast strategy depicted in Fig 3E, and it suggests that including such history-dependent modulation may increase the efficiency of successful source localization.

Since the purely reactive surge-cast model (**Fig 3E**) did not reproduce the early-late distinction we observed in the empirical crossing distribution, we examined the history dependence predicted by two information-accumulation-based theoretical strategies: the centerline-inferring model, based on the model by Grunbaum and Willis [23], and infotaxis, in which movement of a tracking agent explicitly maximizes expected information about the location of the source in 3D space [22]. We found that the centerline-inferring model predicted a history dependence *opposite* to that seen in the data: later crossings generated stronger upwind turns. This inconsistency suggests that accumulated information about a plume’s centerline may not be directly converted into increased upwind advancement along the inferred centerline.

The failure of the centerline-inferring model motivates the consideration of infotaxis, an algorithm in which the tracking agent uses internal knowledge of turbulent plume statistics to interpret odor encounters and guide its search. Though the true plume used both in the original experiments and in our infotaxis simulations was cylindrical and contained within a laminar flow, we nonetheless allowed the simulated agent to assume an internal model of a more natural, plume governed by turbulent diffusion. Essentially, we wanted to ask how a search agent used to turbulence would behave when the plume was actually laminar and cylindrical; this, we felt, best approximated the experimental situation in which flies evolved to track natural plumes were introduced to an artificial one. Additionally, though it is possible the flies in the wind tunnel noticed and adapted to the real plume’s non-turbulent structure, given the high variability of their trajectories and the intermittency of their odor encounters, their dynamic flight and odor experience was likely reasonably similar to that one would expect in more naturalistic plume tracking. Performing similar experiments in the presence of turbulent, yet measurable plumes (measured, for example, using Schlieren imaging, a technique that could detect concentration changes through the optical distortions they cause over a patterned background [30, 31] will be an important step to confirming this hypothesis.

In infotaxis, though the magnitude substantially differed, the *sign* of the observed history dependence was consistent with infotaxis. One possible reason that infotaxis, and perhaps the empirical trajectories, exhibit weaker upwind turns for later encounters is because the physical scale at which information is gained is larger for earlier encounters and smaller for later encounters. That is, the first encounters in a trajectory confine the possible source location to a relatively large region of space upwind of where the encounters occurred, so that it advantages the tracking agent to fly upwind into this space to increase its chances of encountering the source or additional odor puffs. Later encounters, however, provide primarily fine-scale information about the source, since the large-scale location may already be known. Thus, it may behoove the tracking agent to pay more attention to the specific details of where it thinks the source might be, as opposed to flying indiscriminately upwind.

In the experiments we analyzed, the fruit flies tracked ethanol, an attractive odorant that emanates from fruit, and the mosquitoes tracked carbon dioxide, which is produced by mammalian hosts. While we did not investigate plume tracking history dependence in the presence of other odors, if the behaviors we observed were indeed evidence of efficient source localization strategies we would expect to see the same history dependence when flies tracked other odors arising from sources with positive valence. Indeed, for other attractive odors, such as Vector 960 (an odorant used in some fruit fly traps), the reflexive odor-triggered turns are quite similar to those triggered by ethanol [15]. Investigating more detailed plume tracking features as a function of odor identity would be a fruitful topic of future research. For aversive odors, on the other hand, we would not expect the same behavioral responses, as the insects’ goal would presumably no longer be to localize and approach the odor source, and so one would expect different navigation strategies.

### On the role of infotaxis in biological source localization

Although infotaxis was proposed in the context of biological plume tracking, few studies have compared theory to data. In fact, to our knowledge the only study that has directly compared infotaxis to animal behavior was that of Calhoun, Chalasani, and Sharpee [32], in which the authors showed that the foraging behavior of *C*. *elegans* in the absence of a food source exhibited transitions from local to global search strategies similar to those predicted by infotaxis. In addition to the already discussed challenge of comparing theoretical to empirical flight trajectories, infotaxis trajectories bear the challenge of depending on the parameters the tracking agent uses in its turbulent plume model, such as the source emission rate or turbulent diffusivity coefficient. While our qualitative results were relatively invariant to small changes in these parameters, the observed infotaxis history dependence changed sign when the source emission rate assumed by the insect decreased too sharply (**S3 Fig**). *D*, the turbulent diffusivity coefficient assumed by an infotaxis agent in its internal plume model, also strongly affects the source position information gained from each odor detection: with a very small *D*, odor detection implies the source is almost directly upwind, since little spreading should have occurred and which should strongly bias movement in that direction; with a larger *D*, however, odors can be detected from sources with more variable positions. When we re-ran our infotaxis simulations we saw this was indeed the case: smaller *D* led to the predicted stronger upwind turns (**S3 Fig**). The sign of the history dependence, however, which was the key focus of our analysis, did not depend on *D*; for both small and large *D* early plume-crossing-triggered turns had a stronger upwind component than late turns (**S3 Fig**). This shows that while trajectories differ in some ways when the internal plume model changes, certain coarse-resolution features remain the same.

Since an infotaxis agent uses these parameters to infer the source location given a sequence of odor encounters, a key question is how such an agent would learn these parameters in the first place, which can vary over several orders of magnitude in natural environments [33], and incorrectly estimated values of which can affect the efficiency of its search trajectory [34]. One possibility is that by incorporating a reactive component to their behaviors, as observed in the data we analyzed, a tracking strategy might gain robustness to variations in the plume statistics that would otherwise confound a pure infotaxis strategy. That is, a moderately strong reactive component might stabilize behavioral responses that would otherwise be sensitive to the parameters of the plume. Indeed, when plume-tracking robots were programmed with different search strategies, under conditions of high odor concentration, reactive strategies outperformed infotaxis [35], perhaps because of mismatches in the true plume parameters vs. those assumed by the robot. Future work could test this hypothesis explicitly by building a full trajectory-generating model that was a hybrid of infotaxis and a reactive strategy, and calculating source-finding efficiency under varying plume statistics.

### The role of vision in search and infotaxis

An important component of an insect’s search strategy is to explore visual features after encountering attractive odors [15]. Their choice to explore a visual feature likely depends on many factors, which may include the concentration and frequency and number of odor encounters. Thus, it is conceivable that our observed difference in behavior for early and late plume crossings is influenced by the insect’s choice to explore visual features (such as the checkerboard pattern on the floor of the wind tunnel) rather than solely being a function of plume interactions. These same visual features, however, could also help to guide the animal’s surge and cast behavior by providing helpful landmarks.

Of the models we tested, only the infotaxis model captured the correct qualitative change in behavior between early and late plume encounters. The infotaxis model, however, requires that the animal has access to its exact position in world coordinates, something that is quite challenging for an insect flying in the wind over large distances (separating distance, ground speed, and wind speed, from measurements of optic flow and air speed is not trivial [36]. Instead, it possible that insects could use visual features as relative landmarks in an infotaxis-like strategy. We hope that our results will inspire the development of new biologically plausible search theories similar to infotaxis, but that take into account the limited information available to insects, which includes visual landmarks, but not exact location information.

## Materials and methods

All analyses and simulations were written in the Python programming language. All code is available at https://github.com/rkp8000/wind_tunnel.

### Dataset

We used datasets previously described by van Breugel and Dickinson [15] and van Breugel et al. [21]. See the referenced works for more details. Briefly, groups of either fruit flies or mosquitoes were released in a 1.5 m x 0.3 m x 0.3 m wind tunnel (with a 1.3 m x 0.3 m x 0.3 m section available for flight) containing a gentle, laminar wind flowing along the long axis (30–60 cm/s wind speed). When any insect took off, a camera-based tracking system began recording it, and the insect’s 3D trajectory was later reconstructed from the video. In the experimental conditions we have focused on, a laminar odor plume was also present in the wind tunnel (ethanol for fruit fly experiments and CO2 for mosquito experiments). The plume retained a stationary profile due to the laminar wind flow, and was approximately cylindrical in shape, with the highest concentration along the center of the cylinder. The odor concentration as a function of 3D position in the wind tunnel was measured prior to releasing any insects. For the ethanol plume in the fruit fly experiments, there was no discernible widening of the plume from the upwind to the downwind end of the wind tunnel, such that the plume’s cross section did not change along the wind axis. For the CO2 plume some diffusion occurred, with the plume widening slightly with increasing *x*, i.e. towards the downwind end of the wind tunnel. However, since our history dependence analysis accounted for *x* before comparing early vs. late plume crossings, it is unlikely that the trend observed in mosquitoes tracking CO2 was due to perceived changes in the plume cross section. With both plume types, however, the plume’s stationarity allowed the approximate concentration experienced by the insect to be inferred from its position. The resulting dataset that we used as our starting point thus contained registered time-series of experienced odor concentration (up to a multiplicative factor), 3D position and 3D velocity (Kalman filtered with a constant velocity Kalman filter) at every time point in every trajectory of every insect.

### Identifying plume crossings

We defined a plume crossing as the interval during which the odor concentration experienced by the insect rose above a certain threshold. To determine an optimal threshold for each species we asked which threshold best separated potential plume crossings (in which the insect entered the vicinity of the plume but may or may not have crossed it) into below- and above-threshold crossing groups. Specifically, for a range of candidate thresholds, we asked when the difference between the post-crossing headings in the above-threshold group (averaged over crossings and time) was maximally different from that in the below-threshold group. We reasoned that if our chosen threshold were below its true value, the above-threshold group would get diluted with non-crossings, making it more closely resemble the below-threshold group; if our chosen threshold were above the true value, however, then the below-threshold group would get diluted by true crossings, making it more closely resemble the above-threshold group. An optimal threshold that ideally separates the below- and above-threshold groups should exist somewhere in between. However, upon performing this analysis a large uncertainty arose surrounding the calculated difference between the two groups’ heading time series (**S1 Fig**), such that the optimality of the chosen threshold could not be guaranteed. To keep our analysis straightforward, we chose an ethanol threshold approximately corresponding to the “elbows” in **S1A Fig** (for fruit flies) and **S1D Fig** (for mosquitoes), corresponding to 0.0009% ethanol and 430 ppm CO2, respectively, to define plume crossings.

Importantly, however, none of our results depend on the precise threshold used to define a plume crossing, since an additional threshold was either kept as a free parameter (**Fig 2**) or the chief comparison was made between groups defined by the same plume-crossing threshold but which differed along other dimensions, such as crossing history. To provide further evidence that the precise crossing threshold value did not affect our qualitative results, we re-ran the history dependence analysis from **Fig 4** using the 0.3 m/s wind speed fly-tracking-ethanol experiments, but extracting crossing sets according to a range of thresholds that varied over almost two orders of magnitude (**S5 Fig**). We found, however, that regardless of which threshold we chose, the results followed the same trend: late crossings yielded turns with a weaker upwind component than earlier crossings, all else held constant. Additionally, we re-ran our hybrid model analysis using a wide range of thresholds (**S7 Fig**) but also found that this parameter did not substantially affect our results.

In all of our analyses we chose *t* = 0 to correspond to the peak odor concentration experienced during the plume crossing (e.g., as in **Fig 1D**). We defined the end of each plume crossing as the time when the insect either reentered the plume (the experienced concentration rose above threshold again) or landed.

### Data exclusion criteria

In order to limit the influence of geometrical confounds on the results of our analysis, unless otherwise noted we excluded the following sets of plume crossings from all empirical and model-generated datasets when performing our analysis. First, we excluded all crossings occurring in the upwind- or downwind-most 30 cm of the wind tunnel. This was motivated by the possibility that flies already in the upwind end of the wind tunnel may exhibit more crosswind-oriented flight simply because they have less of an area to turn into upwind of them. Second, we excluded all crossings that did not occur at a crosswind angle, i.e., in which the initial crossing heading was not between 60° and 120° from upwind. We made this exclusion simply because we were seeking out upwind directed changes in heading, which would not be visible if flies were already heading upwind. Relaxing these criteria did not change the qualitative results of our analysis of the dataset, but they did introduce confounding factors into our simulated trajectories. For example, when we included the upwind-most 30 cm of the wind tunnel in the surge-cast model analysis (**Fig 3E**), late crossings indeed appeared more crosswind, simply because many late crossings occurred in this upwind-most portion, where the simulated agents’ contacting of the upwind wall often prevented them from turning as upwind as in the earlier crossings that occurred in the central portion of the wind tunnel.

### Partial correlation analysis

To calculate the partial correlation between peak concentration experienced during a plume crossing and the fly’s heading at time *t* following the crossing (**Fig 2**), we first fit a linear model predicting the heading *h*(*t*) from the initial heading *h*_0_ = *h*(0) and the position *x*_0_ along the long axis of the wind tunnel. We then subtracted this prediction from *h*(*t*) to yield a residual heading *h’*(t), and we subsequently calculated the correlation between peak concentration *c_peak_* and *h’*(*t*). Given this framework, any correlations between *c_peak_* and *h’*(*t*) cannot be through the confounding factors of initial heading or position in the wind tunnel.

### Threshold and threshold-linear model fitting

We defined the threshold model as:

$$h(t)=a_{h}h_{0}+a_{x}x_{0}+h_{<}+h_{>}\Theta (c_{peak}-c_{th})$$

where *h*(*t*) is the heading *t* seconds after the plume crossing; *h*_*0*_ is the heading at the time of the plume crossing, scaled by parameter *a*_*h*_; *x*_0_ is the position at the time of the plume crossing along the upwind/downwind axis, scaled by parameter *a*_*x*_; *h*_*<*_ is the heading predicted when the peak crossing concentration *c_peak_* is below the threshold *c*_*th*_; *h*_*>*_ is the additional heading change predicted when the concentration was above *c*_th_, and Θ is the Heaviside step function, which is 0 below 0 and 1 above 0.

We defined the threshold-linear model as:

$$h(t)=a_{h}h_{0}+a_{x}x_{0}+h_{<}+(h_{>}+a_{c}c_{peak})\Theta (c_{peak}-c_{th})$$

where *a*_*c*_ is an additional parameter specifying the linear dependence of heading on concentration when *c_peak_* is greater than the threshold *c*_*th*_.

In both models we fit *a*_*h*_, *a*_*x*_, *h*_*<*_, *h*_*>*_, and *c_th_*. In the threshold-linear model we additionally fit *a*_*c*_. The threshold model is thus “nested” within the threshold-linear model. For both models we chose as the best fit parameters those which minimized the squared difference between the true and the model-predicted headings. To determine whether the threshold-linear model fit the data significantly better, we used an F-test. When calculating the p-value, however, we replaced the number of crossings with the number of unique trajectories, since each trajectory yielded multiple crossings, thus providing a more conservative estimate of significance by not treating all crossings as independent occurrences.

### History dependence analysis

To prevent geometrical factors from confounding the results of our comparison of early vs. late plume crossings, we first constructed a new variable h*(*t*) representing the change in heading at time *t* that remained after linear predictions of Δ*h*(*t*) from *x*_0_ and *t*_*flight*_ (the time spent flying since takeoff) were subtracted from Δ*h*(*t*). In particular, we fit the predictor coefficients and calculated the predictions of Δ*h*(*t*) using all non-excluded plume crossings, regardless of crossing number. Following the calculation of h*(*t*) for each crossing at each time point, we grouped crossings into early and late classes as before and compared h*(*t*) between the two groups using a t-test. Finally, since there were occasionally multiple non-excluded crossings from one trajectory in the same class, when calculating the p-values shown in **Fig 4** we used as the number of data points in each class the number of unique trajectories, as opposed to the number of unique crossings.

In an additional related analysis, also aimed at uncovering a relationship between crossing number and post-crossing heading, instead of splitting crossings into early vs. late crossings we simply calculated the partial correlation between crossing number and Δ*h* time-averaged from 350–450 ms post-crossing. This partial correlation was calculated by first subtracting out the best linear predictions of the time-averaged Δ*h* from *x*_0_ and *t*_*flight*_ (**S4 Fig**).

### Base model for surge-cast and centerline-inferring tracking algorithms

We based both our surge-cast and centerline-inferring algorithms on a correlated random walker, described by:

$$\tau \dot{v}=-v+\eta +bw_{c}$$

where **v** is the tracking agent’s velocity, with the dot indicating its time derivative, *τ* is the timescale of turning, ***η*** is a Gaussian white noise variable sampled independently at each timestep with zero-mean and diagonal covariance matrix with diagonal entries *η*, **w**_c_ is a unit vector in the crosswind plane pointing toward the centerline of the wind tunnel, and *b* is a positive scalar multiplying **w**_c_. Before augmenting the base model to include either surging or centerline-inferring features, we fit *τ*, *η*, and *b* to approximately match the speed, angular velocity, and crosswind position distributions of a simulated agent to those of the empirical data (**S6 Fig**). This yielded values of *τ =* 0.42 s, *η =* 1.9 m/s and *b* = 0.25 m/s. Trajectories generated by the base model alone thus followed a correlated random walk structure with a 0.42 s timescale and with a moderate “casting” bias of oscillating around the wind tunnel centerline.

In both the surge-cast and centerline inferring models, we modeled the wind tunnel to have the same geometry and the plume to have the same concentration profile as in the empirical data. We then analyzed the resulting plume crossings using the same exclusion criteria as we did with the real data.

### Surge-cast model

To add a surging component to the base model just described we augmented it with an additional upwind bias term such that

$$\tau \dot{v}=-v+\eta +bw_{c}+aw_{u}$$

where **w**_u_ is a unit vector pointing upwind, and a(t) is the convolution of an alpha function with timescale 0.07 s and maximum amplitude 0.8 m/s and a train of delta functions placed at the time points corresponding to the peak odor experienced in each crossing. The timescale and amplitude of the alpha function were chosen so that the simulated tracking agent’s plume-crossing triggered responses exhibited approximately the same dynamics as observed in the empirical plume-crossing data.

### Centerline-inferring agent simulation

To generate trajectories for the centerline-inferring algorithm we augmented the base model with a different upwind bias term:

$$\tau \dot{v}=-v+\eta +bw_{c}+kw_{u}.$$

Here *k* is a positive-valued scalar that increases as the uncertainty of the plume’s centerline location decreases. In particular, we let *k* = k*/*|*K|, where k* is the upwind bias and K is the covariance matrix of a posterior belief probability over the 2D centerline location (y*, z*), whose determination is given shortly. Furthermore, in the centerline-inferring agent, we let **w**_c_ be a vector that points not to the centerline of the wind tunnel, but rather to the maximum a posteriori estimate of the centerline of the plume. As with the surge-cast model, we chose the scaling factor for the upwind bias such that the plume-crossing triggered responses showed approximately the same dynamics as in the data.

The 2D belief distribution over the plume centerline location (y*, z*) was updated in a Bayesian way each time the simulated insect crossed the plume at location (*x*_*n*_, *y*_*n*_, *z*_*n*_) (with *n* indexing the plume crossing number):

$$P(y^{*},z^{*}|y_{n},z_{n},y_{n-1},z_{n-1},\ldots )\;\alpha \;P(y_{n},z_{n}|y^{*},z^{*},y_{n-1},z_{n-1})P(y^{*},z^{*}|y_{n-1},z_{n-1},\ldots )$$

We assumed that the likelihood and prior (the two terms on the right-hand side of the equation, respectively) were both Gaussian with diagonal covariances. Thus, the posterior (the term on the left-hand side of the equation), the likelihood, and the prior could each be parameterized by a mean and covariance matrix. Note that in this model the posterior after one crossing acts as the prior at the next crossing. We chose the likelihood covariance *K*_*s*_ and the covariance *K*_*0*_ of the prior over (y*, z*) before any crossings had occurred to have diagonal entries (2m)^2^ and (5m)^2^, respectively. Finally, to model short-term memory decay over several seconds, we allowed the mean of the posterior to decay exponentially to (0, 0) over *τ*_*m*_ = 10 seconds, with the posterior covariance *K* decaying to *K*_*0*_ over the same timescale.

### Infotaxis simulation

The infotaxis algorithm we used was developed by and detailed in [22]. In this algorithm a tracking agent moves along a rectangular lattice in order to localize the source of an odor plume. To model turbulence, the odor plume is not assumed to have a concentration gradient, but rather to yield random “hits”, with hit probability maximized immediately downwind of the source. The tracking agent maintains a belief distribution over the possible source locations that is updated at each time step, depending on whether the agent receives a hit or a miss. This update is Bayesian and thus depends on an explicit formula for the probability of receiving a hit a function of displacement from the source, that is, the likelihood. The principle difference between the Bayesian update in the centerline-inferring model vs. in infotaxis is that in the centerline-inferring model the belief distribution is a 2D Gaussian over the centerline location, whereas in infotaxis the distribution is over the 3D source position coordinate and has no parametric form. Since hit probability depends on the agent’s position, and hits and misses update the source position distribution in different ways, the tracking agent moves so as to maximize the expected decrease in uncertainty of the source position distribution, as measured by its Shannon entropy.

The likelihood formula is derived in [22] from the time-averaged hit rate one would expect at different displacements from a source in a turbulent medium. Importantly, this hit rate depends on *D*, the effective diffusivity of the turbulent medium [24]. When *D* is small, the plume is confined to a narrow band downwind of the source, and when *D* is large the plume becomes much more spread out, and can even yield hits upwind of the source.

We ran our simulations on a 3D lattice that simulated positions accessible within the wind tunnel, with lattice points 2 cm apart from one another. A discretized version of the non-turbulent cylindrical plume used in the experiments was simulated in the center of the lattice, and the tracking agent received a hit at every time step it was inside the plume. Note, however, that even though the simulated plume was cylindrical and non-turbulent, the actions of the tracking agent in our simulation were driven by its assumption that it was interacting with a naturalistic, turbulent plume. For each experimental flight trajectory we generated one corresponding infotaxis trajectory that started at the same (but discretized) location in the wind tunnel as the experimental trajectory and which lasted for the same number of timesteps as the number of lattice points that the experimental trajectory traversed.

## Supporting information

S1 Fig Determination of concentration thresholds for each experiment. The thick line shows the difference between the mean plume-crossing-triggered heading time-series for crossings above the threshold and the mean plume-crossing-triggered heading time-series for crossings below the threshold, time-averaged over the first one second following the plume crossing. Shading represents uncertainty, calculated by propagating the standard errors of the means of each group through the difference calculation. Each panel corresponds to a different experiment, labeled by the insect and the wind speed.
(TIFF)

S2 Fig Positional differences between early and late plume crossings. Each panel shows the distribution of the upwind/downwind components of plume-crossing positions for either the early or late groups shown in **Fig 3**, measured at the time of the plume crossing. As in **Fig 3**, we have excluded all crossings occurring in the most upwind or most downwind 30 cm of the wind tunnel (leaving 70 cm of valid flight space for our analysis) and of the remaining crossings we have included only those in which the heading at the time of the crossing was between 60 and 120 degrees. Each panel corresponds to one insect/wind-speed, with the difference in the mean plume-crossing upwind/downwind position (x) shown in meters in the title.
(TIFF)

S3 Fig Infotaxis history dependence for varied plume estimate parameters. Same layout as in **Fig 3E–3H**, but with different infotaxis parameters. In A-B, the source emission rate R is 1000Hz, and the turbulent diffusivity coefficient D is varied, as indicated in the panel titles. In C-E, the turbulent diffusivity coefficient D is 0.09 m^2^/s, with R varied, as indicated in the panel titles.
(TIFF)

S4 Fig Partial correlation between heading change and crossing number, conditioned on x_0_ and t_flight_. Each panel corresponds to a different experiment. The x-axis shows x_0_ the x-position at the time of the crossing, and the y-axis shows the change in heading, time-averaged from 350 to 450 ms post-crossing. Each point corresponds to one crossing, with the color of the points denoting the crossing number (“cn”; crossing numbers of 1 or 2 correspond to early crossings and 3, 4, 5 to late crossings; we did not include the small number of crossings with crossing number > 5). The partial correlation coefficients between crossing number and the Δheading, conditioned on x_0_, and the corresponding p-values, are shown in the panel titles. When calculating p-values, we used the number of unique trajectories, rather than the number of crossings, since one trajectory frequently contained multiple crossings.
(TIFF)

S5 Fig Effects of varying odor detection threshold on history dependence analysis. In each panel the analysis from **Fig 4** is performed on a set of plume crossings where crossings are defined as trajectory portions in which the odor concentration rises at least once above a minimum detection threshold (varying by panel and listed in panel titles). All panels show crossings calculated from trajectories in which flies were tracking ethanol in a 0.3 m/s wind speed. As crossing-detection threshold varies over more than an order of magnitude, the key history dependent features remain qualitatively constant.
(TIFF)

S6 Fig Distributions of kinematic quantities in base model (before surge-cast or centerline-inferring features were added) vs. empirical data. Each panel shows the distribution of speeds (A), angular velocities (B), or crosswind positions (C) (calculated across all time point) of the empirical trajectories (black) vs. the trajectories generated by best-fit base model for the surge-cast and centerline-inferring models. Y-axis units are arbitrary and denote relative proportions of time points.
(TIFF)

S7 Fig Effects of varying odor detection threshold on hybrid model analysis. Here we show the optimal surge-cast percentage in the surge-cast-infotaxis hybrid crossing model analysis introduced in **Fig 6** as a function of the odor detection threshold used to generate and extract crossings from the trajectories. The points and error bars show the mean and standard deviation of the threshold-dependent distributions equivalent to **Fig 6C**.
(TIFF)

S8 Fig Position distributions for data and infotaxis results for different wind speeds. Equivalent to **Fig 6A and 6D**. A, B show position distributions for empirical data and infotaxis simulations in a wind tunnel with 0.4 m/s wind speeds, respectively. C, D show the same for a 0.6 m/s wind speed.
(TIFF)
