# Supplementary figures and images for: History dependence in insect flight decisions during odor tracking
Source: PLoS Comput Biol. 2018 Feb 12;14(2):e1005969. doi: 10.1371/journal.pcbi.1005969 (PMC5828511; doi:10.1371/journal.pcbi.1005969)

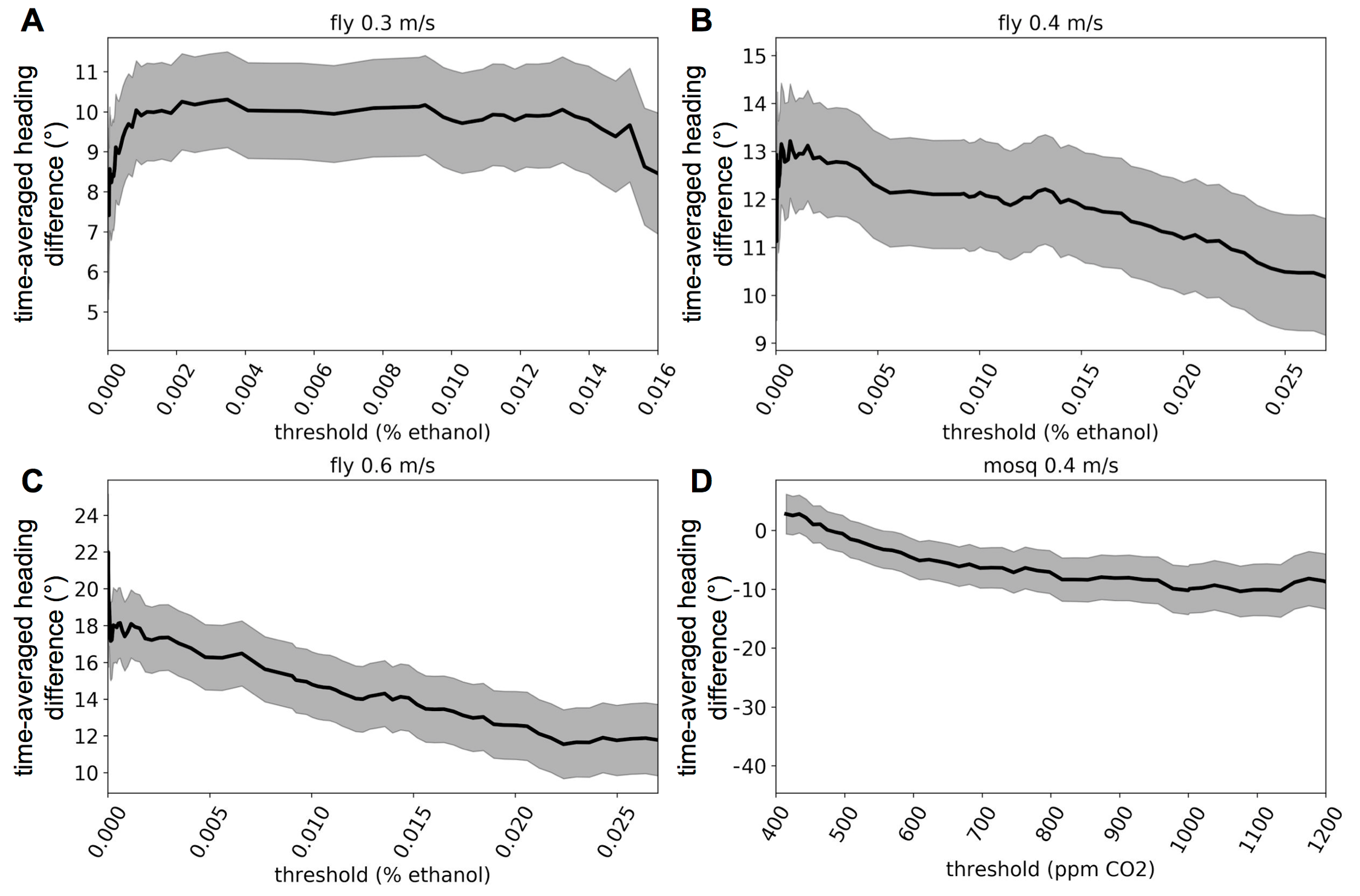

Supplement: S1 Fig — The thick line shows the difference between the mean plume-crossing-triggered heading time-series for crossings above the threshold and the mean plume-crossing-triggered heading time-series for crossings below the threshold, time-averaged over the first one second following the plume crossing. Shading represents uncertainty, calculated by propagating the standard errors of the means of each group through the difference calculation. Each panel corresponds to a different experiment, labeled by the insect and the wind speed. (TIFF) [file pcbi.1005969.s001.tiff]

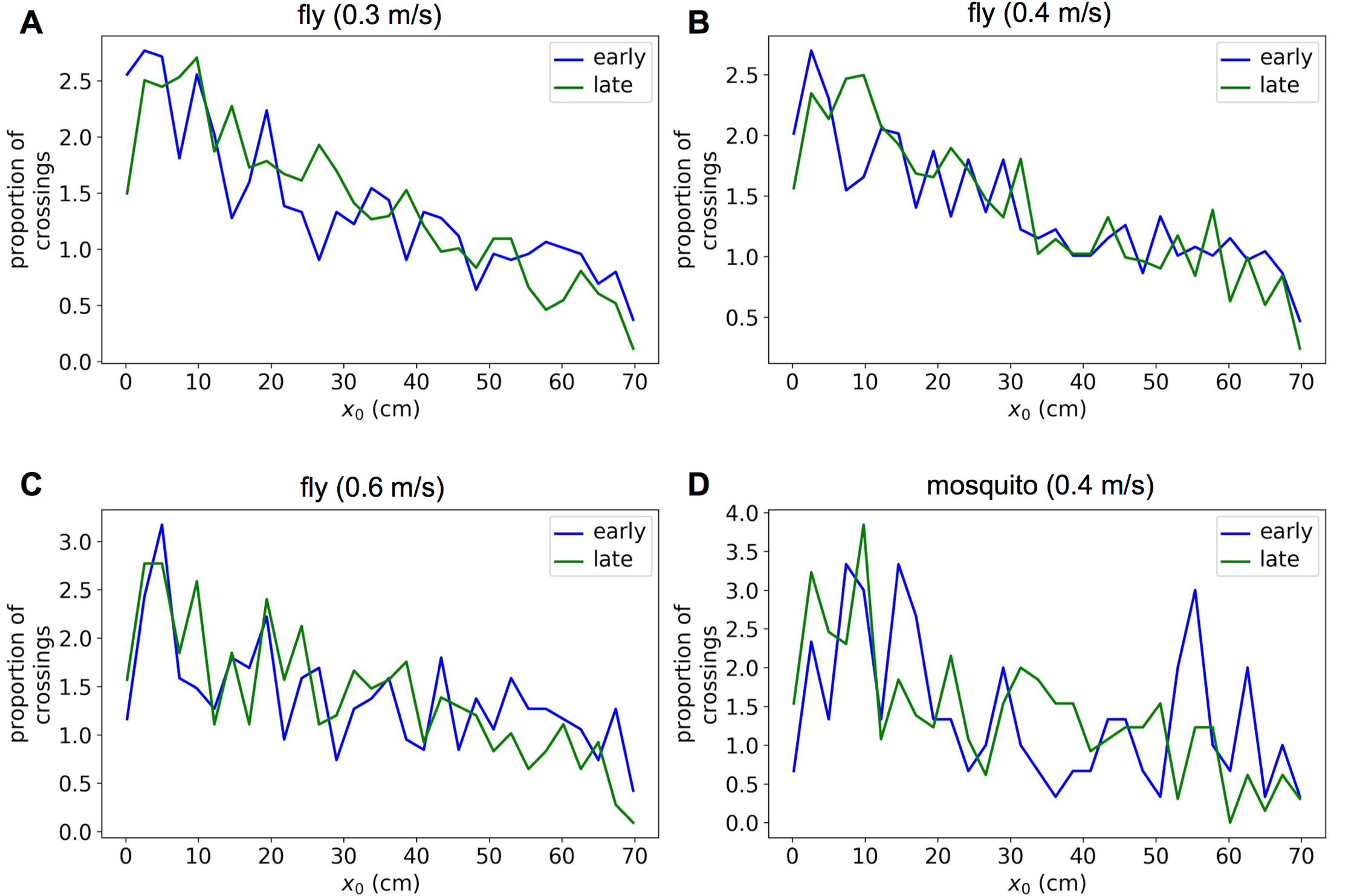

Supplement: S2 Fig — Each panel shows the distribution of the upwind/downwind components of plume-crossing positions for either the early or late groups shown in Fig 3, measured at the time of the plume crossing. As in Fig 3, we have excluded all crossings occurring in the most upwind or most downwind 30 cm of the wind tunnel (leaving 70 cm of valid flight space for our analysis) and of the remaining crossings we have included only those in which the heading at the time of the crossing was between 60 and 120 degrees. Each panel corresponds to one insect/wind-speed, with the difference in the mean plume-crossing upwind/downwind position (x) shown in meters in the title. (TIFF) [file pcbi.1005969.s002.tiff]

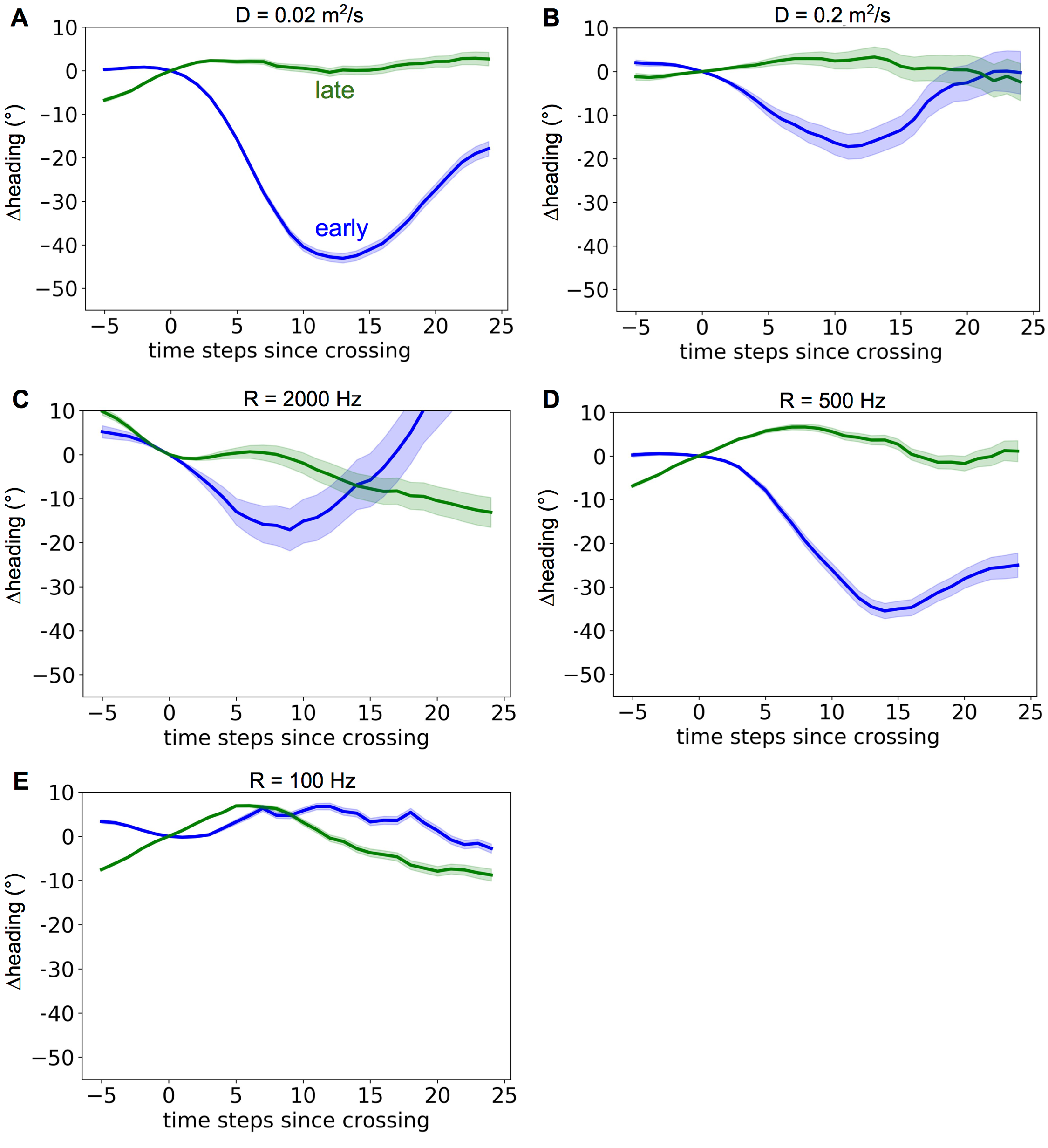

Supplement: S3 Fig — Same layout as in Fig 3E–3H, but with different infotaxis parameters. In A-B, the source emission rate R is 1000Hz, and the turbulent diffusivity coefficient D is varied, as indicated in the panel titles. In C-E, the turbulent diffusivity coefficient D is 0.09 m2/s, with R varied, as indicated in the panel titles. (TIFF) [file pcbi.1005969.s003.tiff]

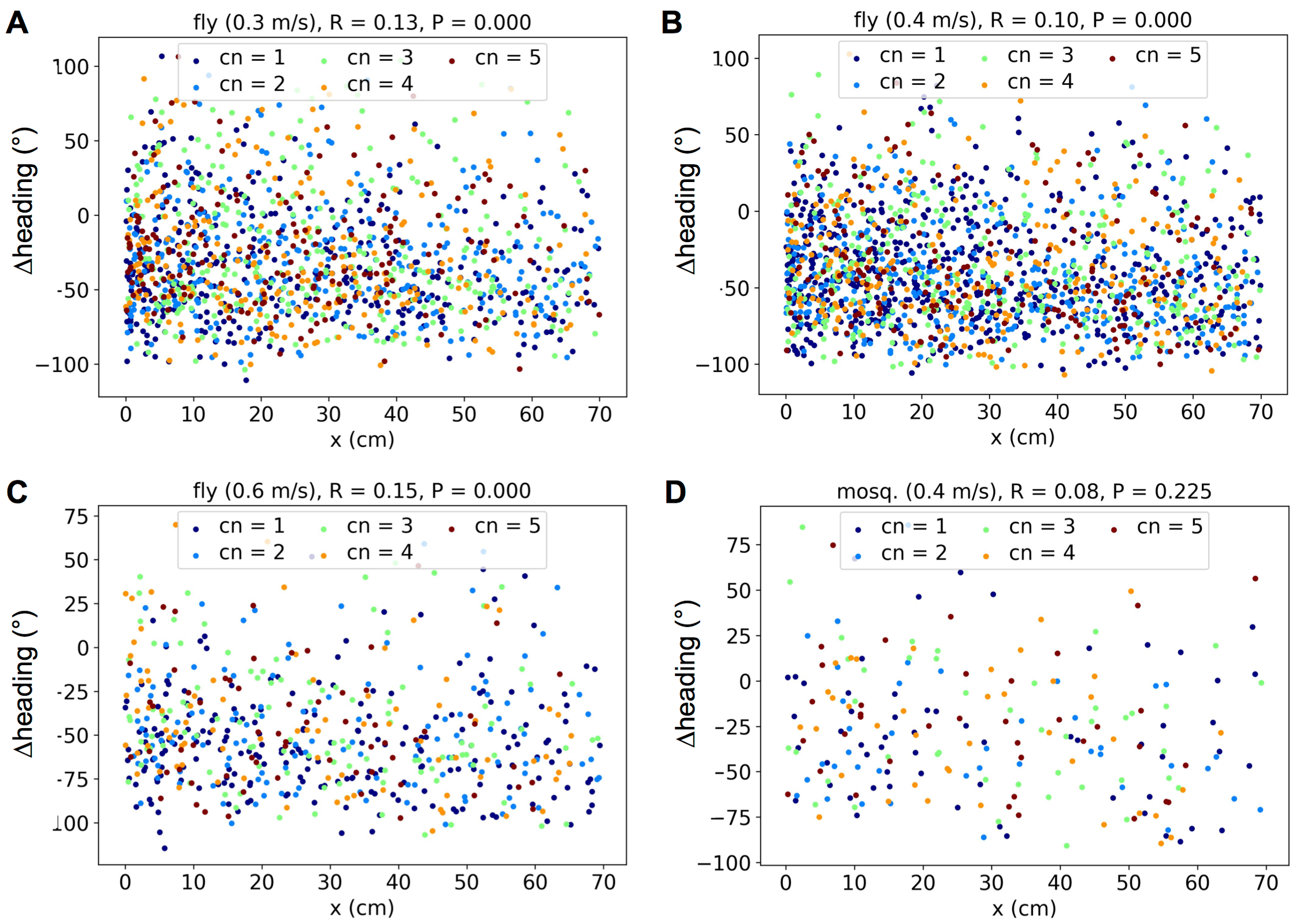

Supplement: S4 Fig — Each panel corresponds to a different experiment. The x-axis shows x0 the x-position at the time of the crossing, and the y-axis shows the change in heading, time-averaged from 350 to 450 ms post-crossing. Each point corresponds to one crossing, with the color of the points denoting the crossing number (“cn”; crossing numbers of 1 or 2 correspond to early crossings and 3, 4, 5 to late crossings; we did not include the small number of crossings with crossing number > 5). The partial correlation coefficients between crossing number and the Δheading, conditioned on x0, and the corresponding p-values, are shown in the panel titles. When calculating p-values, we used the number of unique trajectories, rather than the number of crossings, since one trajectory frequently contained multiple crossings. (TIFF) [file pcbi.1005969.s004.tiff]

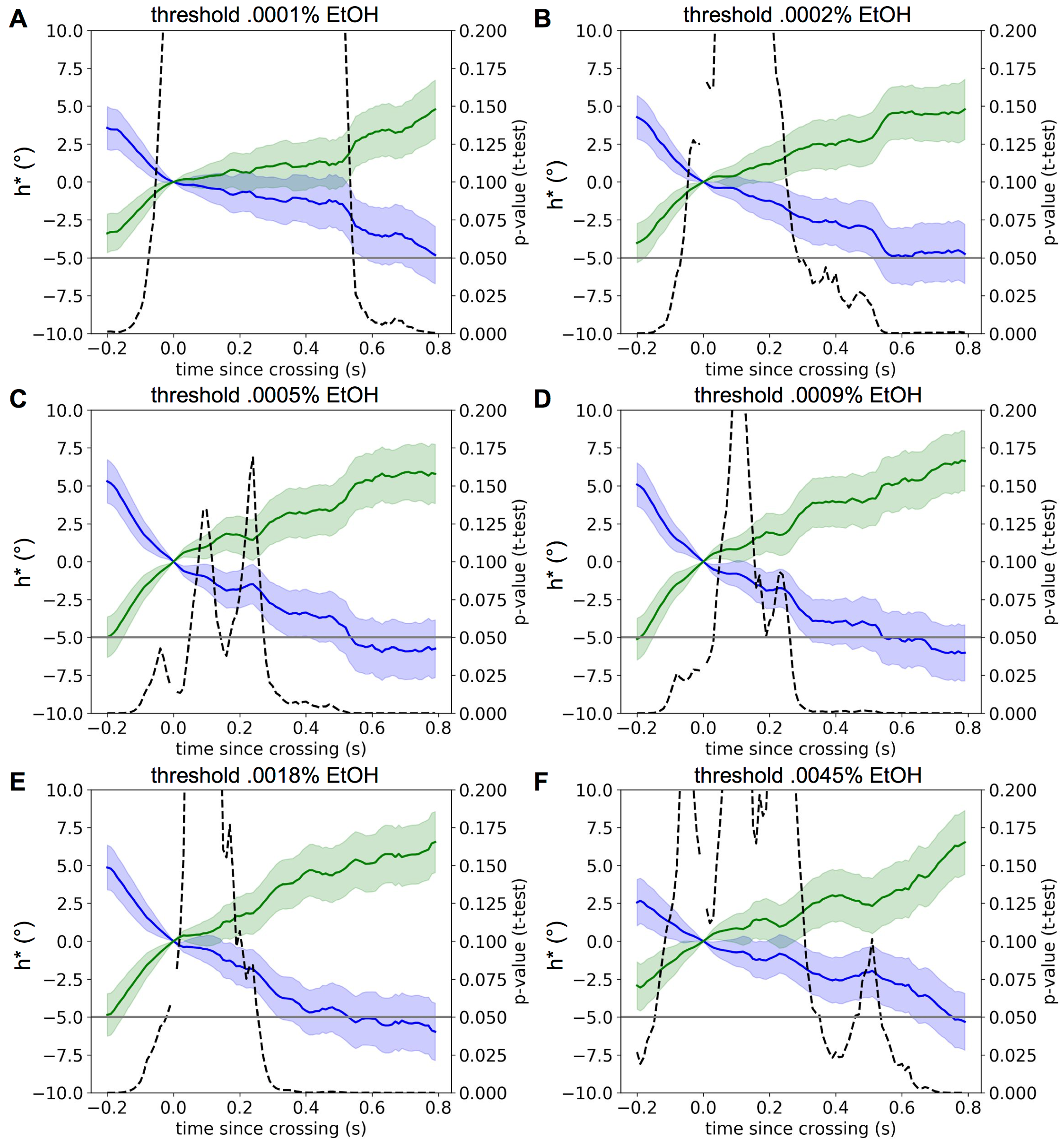

Supplement: S5 Fig — In each panel the analysis from Fig 4 is performed on a set of plume crossings where crossings are defined as trajectory portions in which the odor concentration rises at least once above a minimum detection threshold (varying by panel and listed in panel titles). All panels show crossings calculated from trajectories in which flies were tracking ethanol in a 0.3 m/s wind speed. As crossing-detection threshold varies over more than an order of magnitude, the key history dependent features remain qualitatively constant. (TIFF) [file pcbi.1005969.s005.tiff]

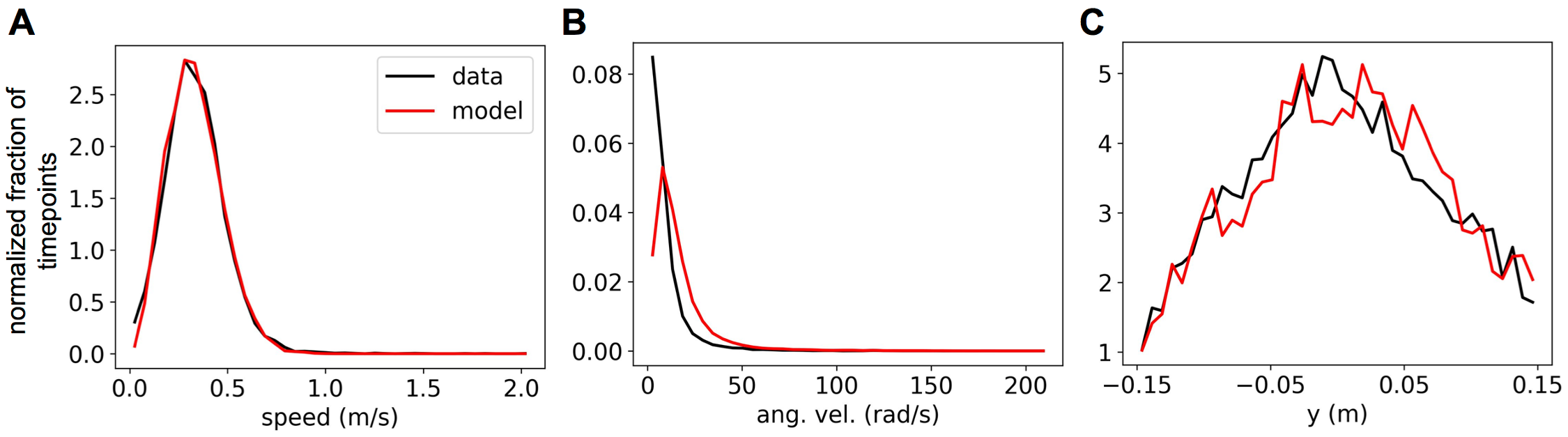

Supplement: S6 Fig — Each panel shows the distribution of speeds (A), angular velocities (B), or crosswind positions (C) (calculated across all time point) of the empirical trajectories (black) vs. the trajectories generated by best-fit base model for the surge-cast and centerline-inferring models. Y-axis units are arbitrary and denote relative proportions of time points. (TIFF) [file pcbi.1005969.s006.tiff]

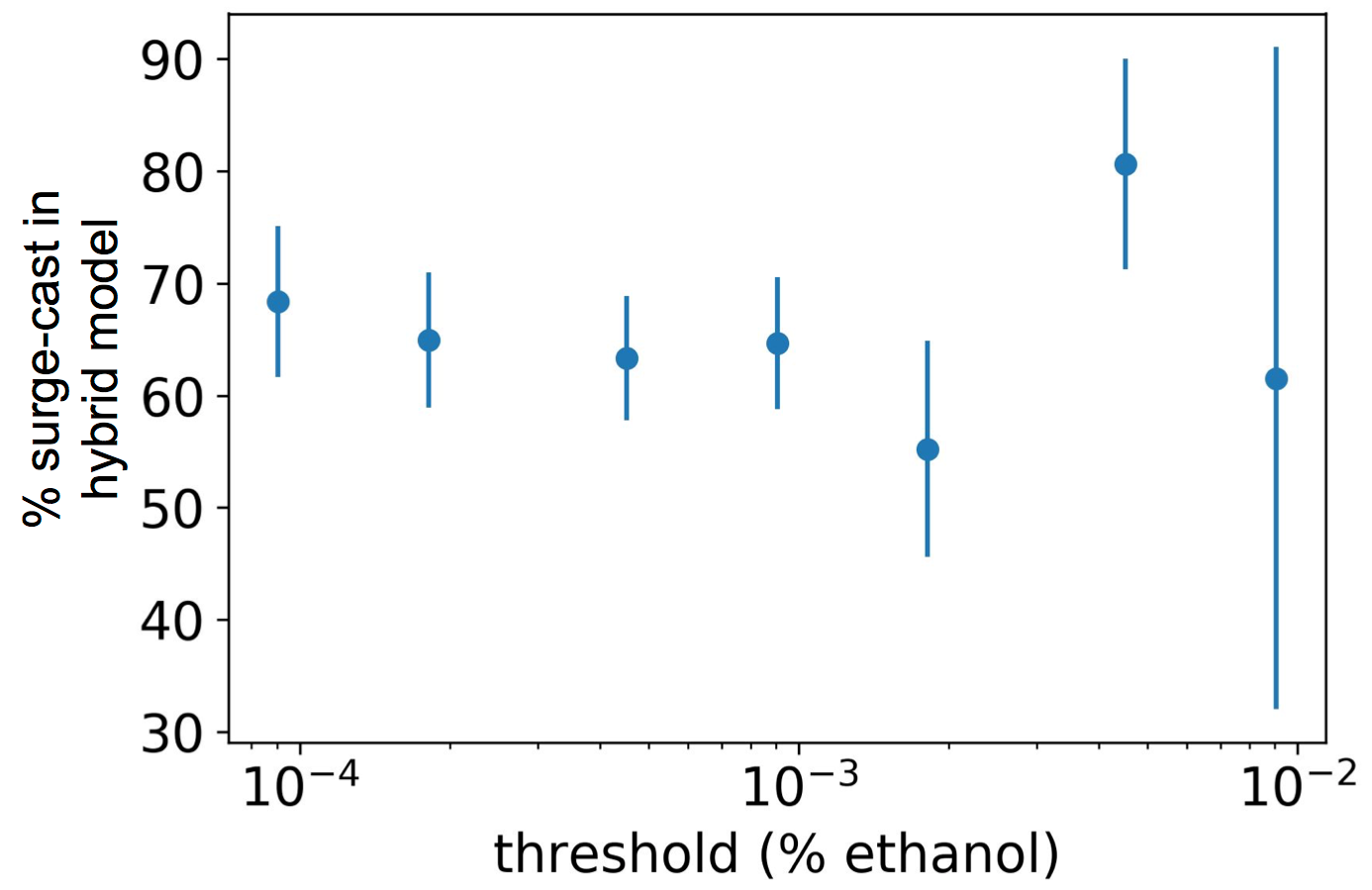

Supplement: S7 Fig — Here we show the optimal surge-cast percentage in the surge-cast-infotaxis hybrid crossing model analysis introduced in Fig 6 as a function of the odor detection threshold used to generate and extract crossings from the trajectories. The points and error bars show the mean and standard deviation of the threshold-dependent distributions equivalent to Fig 6C. (TIFF) [file pcbi.1005969.s007.tiff]

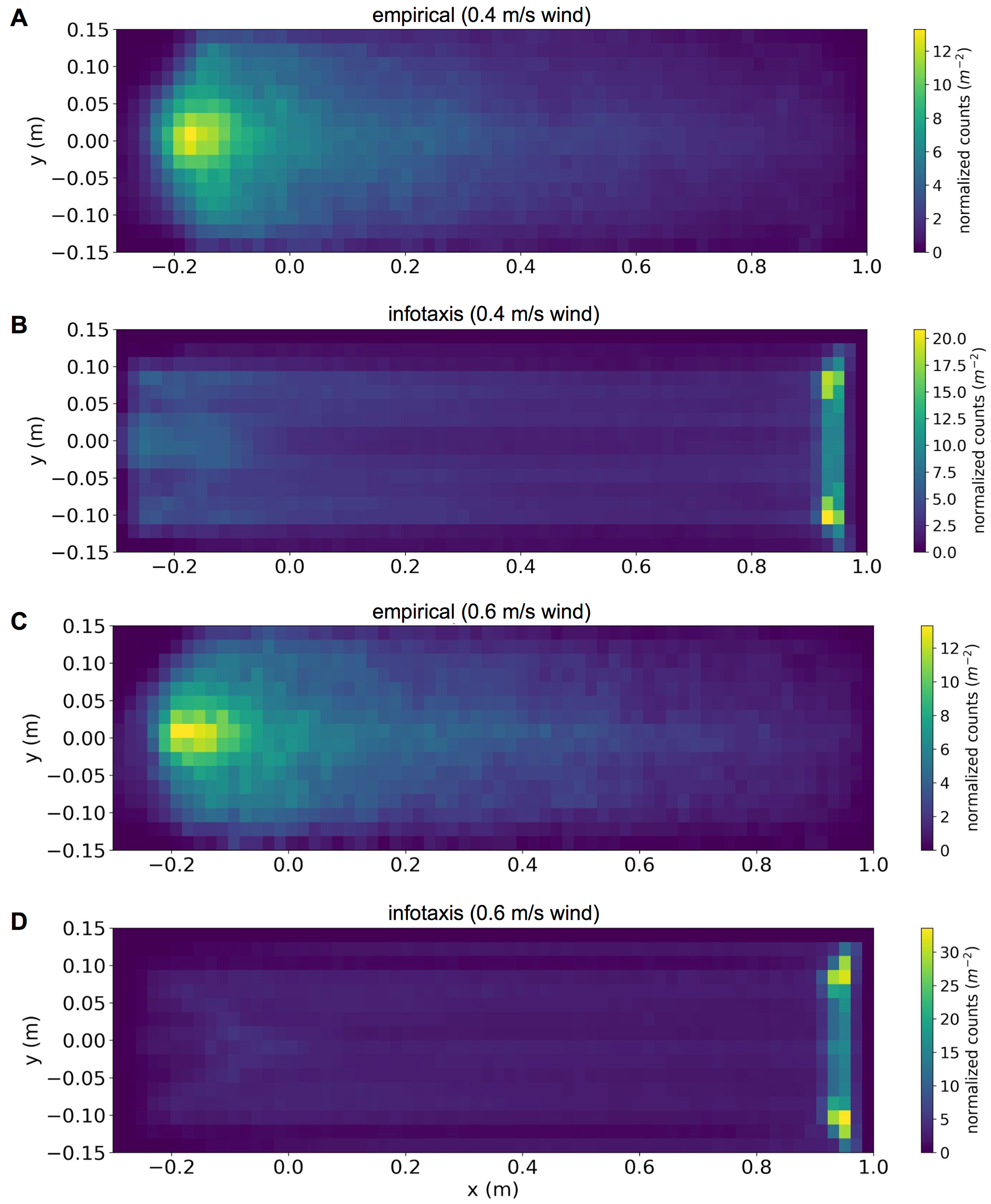

Supplement: S8 Fig — Equivalent to Fig 6A and 6D. A, B show position distributions for empirical data and infotaxis simulations in a wind tunnel with 0.4 m/s wind speeds, respectively. C, D show the same for a 0.6 m/s wind speed. (TIFF) [file pcbi.1005969.s008.tiff]
